# Health outcomes associated with reallocations of time between sleep, sedentary behaviour, and physical activity: a systematic scoping review of isotemporal substitution studies

**DOI:** 10.1186/s12966-018-0691-3

**Published:** 2018-07-13

**Authors:** Jozo Grgic, Dorothea Dumuid, Enrique Garcia Bengoechea, Nipun Shrestha, Adrian Bauman, Timothy Olds, Zeljko Pedisic

**Affiliations:** 1 Institute for Health and Sport (IHES), Victoria University, Melbourne, Australia; 2 Department of Physical Education and Sport Sciences, Faculty of Education and Health Sciences, University of Limerick, Limerick, Ireland; 3 Alliance for Research in Exercise, Nutrition and Activity (ARENA), School of Health Sciences, University of South, Adelaide, Australia; 4 Prevention Research Collaboration, School of Public Health, Sydney University, Sydney, NSW Australia

## Abstract

**Background:**

During a 24-h day, each given period is spent in either sedentary behaviour, sleeping, light physical activity (LPA), or moderate-to-vigorous physical activity (MVPA). In epidemiological research most studies have traditionally analysed the associations of these behaviours in isolation from each other; that is, without taking into account the displacement of time spent in the remaining behaviours. In recent years, there has been a growing interest in exploring how all the behaviours across the energy expenditure spectrum influence health outcomes. A statistical model used to investigate these associations is termed an isotemporal substitution model (ISM). Considering the increasing number of ISM-based studies conducted in all age groups, the present paper aimed to: (i) review and summarise findings from studies that employed ISM in sleep, sedentary behaviour, and physical activity research; (ii) appraise the methodological quality of the studies; and (iii) suggest future research directions in this area.

**Methods:**

A systematic search of ten databases was performed. The Newcastle–Ottawa scale was used to assess the methodological quality of the included studies.

**Results:**

Fifty-six studies met the inclusion criteria, all being of moderate or high methodological quality. Associations were reported for exchanged time varying from one minute to 120 min/day across the studies, with 30 min/day being the most common amount of time reallocated. In total, three different ISM methodologies were used. The most commonly studied health outcomes in relation to isotemporal substitutions were mortality, general health, mental health, adiposity, fitness, and cardiometabolic biomarkers. It seems that reallocations of sedentary time to LPA or MVPA are associated with significant reduction in mortality risk. Current evidence appears to consistently suggest that reductions in mortality risk are greater when time spent sedentary is replaced with higher intensities of physical activity. For adiposity, it seems that reallocating sedentary time to physical activity may be associated with reduced body mass index, body fat percentage, and waist circumference in all age groups, with the magnitude of associations being greater for higher intensities of physical activity. While there is a relatively large body of evidence reporting beneficial associations between the reallocation of time from sedentary behaviour to LPA or MVPA and cardiometabolic biomarkers among adults, there is a lack of studies among children, adolescents, and older adults. Although some studies investigated general health, mental health, and fitness outcomes, further investigation of these topics is warranted. In general, it seems that the strongest association with health outcomes is observed when time is reallocated from sedentary behaviour to MVPA. Most studies did not account for sleep time, which is a major limitation of the current evidence.

**Conclusions:**

The current evidence indicates that time reallocation between sleep, sedentary behaviour, LPA, and MVPA may be associated with a number of health outcomes. Future studies should employ longitudinal designs, take into account all movement behaviours, and examine a wider range of health, psychological, social, economic, and environmental outcomes.

**Electronic supplementary material:**

The online version of this article (10.1186/s12966-018-0691-3) contains supplementary material, which is available to authorized users.

## Background

During a 24-h day, each given period is spent in either sedentary behaviour, sleeping, light-intensity physical activity (LPA), or moderate-to-vigorous physical activity (MVPA) [1, 2]. It has been well documented that all of these time-use components across a 24 h spectrum may be significantly associated with health. For instance, an increased risk of all-cause mortality, cardiovascular disease, metabolic syndrome, type 2 diabetes mellitus (T2D), and certain types of cancer is associated with low levels of MVPA, large amounts of time spent in sedentary behaviour and inappropriate sleep duration [1–10]. As the duration of a day is fixed and finite, a change in one of these movement-related behaviours will result in a net equal and opposite change in other behaviours. Despite this fact, most previous epidemiological studies have analysed the associations of each behaviour in isolation; that is, without taking into account the displacement of the remaining co-dependent behaviours.

In recent years, there has been growing interest in exploring how all the behaviours across the energy expenditure spectrum influence health outcomes [1, 2, 11–15]. In the seminal work by Mekary et al. [12], the isotemporal substitution model (ISM) was proposed as a method for evaluating the displacement of one movement-related behaviour time-use component with another, while allowing adjustment for the confounding effect of the remaining time-use components [12]. In addition to the ISM proposed by Mekary et al. [12], Chastin et al. [13] and Dumuid et al. [14] recently introduced two different compositional isotemporal substitution models that account for the compositional properties of time-use data [1, 2, 15]. The findings of studies employing ISM may improve our understanding of the interrelationships between different movement-related behaviours and their relationships to health and may also help in shaping public health guidelines and promotion strategies [11]. Whilst, for example, most public health guidelines on physical activity recommend people to engage in a certain amount of MVPA, they lack an instruction on which movement/non-movement behaviour should preferably be displaced by MVPA [16, 17]. Studies using ISM may enable creating specific, evidence-based recommendations on favourable reallocations of time between sleep, sedentary behaviour, and physical activity, which has the potential to improve the translation of research findings on these behaviours into practice, and, consequently, increase the uptake of public health messages.

A recent meta-analysis of five studies using ISM concluded that reallocating sedentary time to MVPA was significantly associated with a reduction in percentage of body fat [18]. However, the review focused only on studies conducted among children, with markers of adiposity being the only outcome of interest. An increasing number of ISM-based studies conducted in all age groups have recently been published, investigating outcomes such as adiposity, metabolic biomarkers, mental health, chronic musculoskeletal pain, fitness, mortality, and health-related quality of life [19–39].

A comprehensive review on the topic of time reallocation and its associations on health outcomes seems warranted, to summarise the current state of knowledge in this growing field and provide directions for its future development. Therefore, the aim of this paper was threefold: (i) to review and summarise findings from studies that employed ISM in sleep, sedentary behaviour, and physical activity research; (ii) appraise the methodological quality of the studies; and (iii) suggest future research directions.

## Methods

### Protocol

The methods employed in this review were registered in advance in the PROSPERO register of systematic reviews (ref: CRD42017071606). The Preferred Reporting Items for Systematic Reviews and Meta-Analyses guidelines were followed in this systematic scoping review [40].

### Search strategy

A systematic search of the following databases was performed in July 2017: Academic Search Premier, CINAHL, Health Source: Nursing/Academic Edition, MasterFILE Premier, PsycINFO, PubMed/MEDLINE, Scopus, SPORTDiscus, and Web of Science (including Arts & Humanities Citation Index, Conference Proceedings Citation Index- Science, Conference Proceedings Citation Index- Social Science & Humanities, Science Citation Index Expanded, and Social Sciences Citation Index). The following keywords were used for the search: “physical activity”, “physical inactivity”, sedentar*, sleep*, sitting, standing, isotemporal, compositional. The terms used for the search were combined with Boolean operators (Additional file 1). No limitations regarding publication date were applied. The search results were downloaded and scrutinized in the EndNote software X8 (Clarivate Analytics, New York, USA). A secondary search was performed by: (i) screening the reference lists of each read full-text; and (ii) by performing forward citation tracking of the included studies through Google Scholar and Scopus.

### Inclusion criteria

To be included in the current review, studies were required to meet the following criteria: (i) the study was published in an English-language refereed journal with full-text availability; (ii) the study was an original research (reviews were not considered) with data collected among human participants; (iii) the study used ISM to explore the association between reallocating relevant movement-related behaviours (e.g., sleep, sedentary behaviour, LPA and/or MVPA) and health outcomes. The World Health Organisation [41] defines health as the absence of disease and frailty, as well as complete physical, mental, and social well-being. For the purpose of this paper, any analysed outcome aligned with this definition was considered a health outcome. Observational studies conducted in any age group were considered.

### Study coding and data extraction

The information extracted from the included studies comprised descriptive data including: the geographic location of the study; study design; sample size; measures of sleep, sedentary behaviour, LPA, and MVPA; the ISM used; amount of reallocated time; adjustments for confounding; and the main findings related to the health outcome(s). The data were extracted and tabulated to an Excel spreadsheet predefined for the purpose of this review.

### Methodological quality appraisal

In order to assess the methodological quality of the studies that met the inclusion criteria we used the Newcastle–Ottawa quality assessment scale for observational studies [42]. The scale details can be found elsewhere [42]. The maximum score on the scale was eight. Studies that scored more than six points were considered of high quality, studies scoring 4–6 points were considered as moderate quality and studies with scores less than four points were considered as being of low methodological quality [43].

The search and methodological quality appraisal was performed independently by two authors of the review (JG and EGB). In addition, two authors (JG and NS) independently performed the data extraction. Any discrepancies between the reviewers were resolved with discussion and consensus or in consultation with a third investigator (ZP).

## Results

### Study selection

**Fig. 1:**
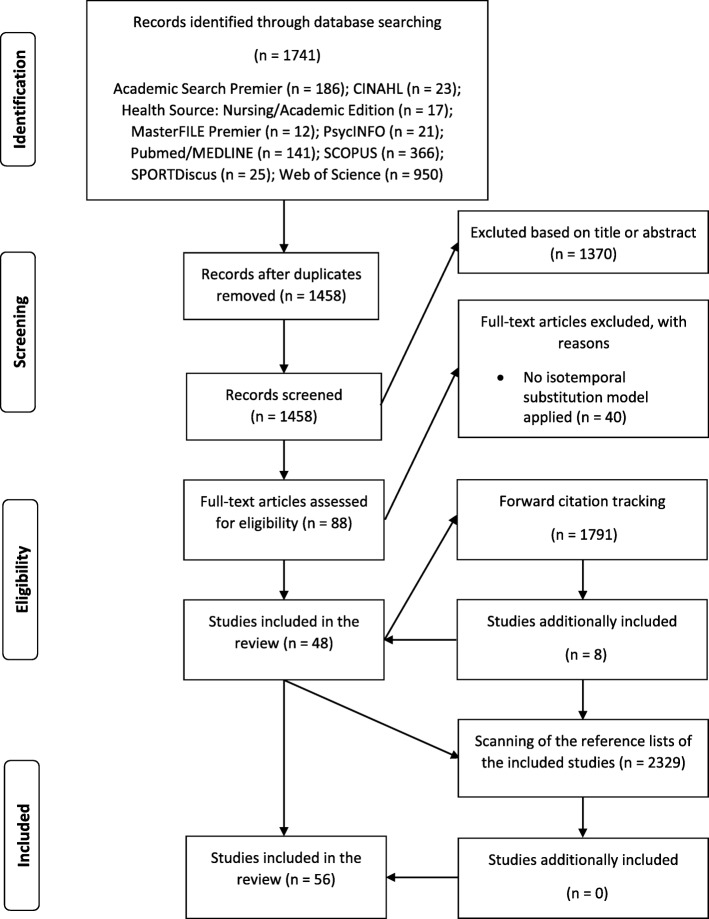
Flow diagram of the search process

In total, 5859 items were screened in the study selection process. The initial database search identified 1741 results. For 88 studies the full-text was retrieved and assessed for eligibility. Of those, 48 studies met the inclusion criteria. Forward citation tracking of the included studies yielded another 1789 search results, of which eight studies were included. By screening through the reference lists of all the included studies (2329 results) we identified no additional studies relevant for this review. Therefore, the total number of studies included in this review is 56 [12, 13, 19–39, 44–76]. The search and study selection process is depicted in Fig. 1.

### Study characteristics

Of the 56 included studies, 36 had a cross-sectional design while 18 were prospective cohort studies. Two studies reported both cross-sectional and prospective relationships. Most of the studies were performed in the USA (*n* = 21), eleven studies were performed in the UK, five in Australia, four in Sweden, three in the Netherlands, two in Canada and Spain, and one in Portugal, Norway, Japan, Greenland, Finland, China, and Denmark. One study used data from Brazil, Europe, and the USA. Accelerometers were used in 41 studies, self-reports were used in 12 studies, while heart rate and other movement monitors were used in three studies. Most studies did not include all the relevant daily or 24-h movement-related behaviours. For example, sleep duration was included in only 22 studies. Associations were reported for exchanged time varying from one minute to 120 min/day across the studies, with 30 min/day being the most common reallocation. The model proposed by Mekary et al. [12] was used in 53 studies. Two studies used the model proposed by Chastin et al. [13], and one study used the model proposed by Dumuid et al. [14].

### Participants and outcomes

The sample sizes of the included studies ranged from 87 to 423,659 participants. The median number of participants per study was 1497. Studies were performed in children and youth [20, 21, 23, 25, 27, 28, 45, 49, 59, 60, 63, 64, 70], adults [12, 13, 22, 24, 30–33, 35, 36, 38, 39, 46, 48, 50–52, 54–56, 58, 62, 65–69, 71, 72, 75, 76], older adults [26, 29, 45, 48, 61] and clinical populations [19, 34, 53, 57, 73, 74]. The outcomes were categorised into six major categories: mortality (*n* = 9 studies; Table 1), perceived health (*n* = 6 studies; Table 2), mental health (*n* = 3 studies; Table 3), adiposity (*n* = 29 studies; Table 4), fitness (*n* = 8 studies; Table 5), cardiometabolic biomarkers (*n* = 18 studies; Table 6) and chronic diseases and conditions (*n* = 6 studies; Table 7). Some of the studies assessed multiple outcomes, therefore, the total number of exceeds 56.

**Table 1:** Summary of the findings from the studies assessing mortality outcomes

Study	Sample and study design	Measures of sleep, SB, LPA, MVPA	Outcome measures	Data analysis method/reallocated time	Adjustments for confounding	Results
Chomistek et al. [50]	Men (*n* = 44,551) from The Health Professionals Follow-up Study, USA; prospective cohort	LPA, MPA, VPA – self report; sleep, SB – not assessed	Sum of total CVD risk factors, total cancer, or other nontraumatic death.	Mekary et al. [12]/10 MET h-wk^− 1^	Smoking, aspirin use, vitamin E supplement use, parental history of myocardial infarction or cancer, alcohol consumption, energy-adjusted intake of polyunsaturated fat, trans fatty acids, omega-3 fatty acids, fiber, diabetes, hypertension, and hypercholesterolemia.	HR (95% CI) MPA → VPA: 0.96 (0.93, 0.99)
Fishman et al. [54]	Adults (*n* = 3029) from the 2003–2004 and 2005–2006 waves of the National Health and Nutrition Examination Survey, USA; prospective cohort	SB, LPA, MVPA – waist-worn accelerometers; sleep – not assessed	Mortality risk	Mekary et al. [12]/10, 30 and 60 min	Age, sex, race/ethnicity, education, and minutes of device wear time (model 1). Model 2 includes BMI, smoking, the presence of diabetes, coronary heart disease, congestive heart failure, stroke, cancer, and mobility limitation. In model 3 those with prevalent chronic illnesses (diabetes, coronary heart disease, congestive heart failure, stroke, and cancer) or mobility limitations at baseline were excluded.	HR (95% CI) 10 min reallocation Model 1 SB → LPA: 0.90 (0.88, 0.93) SB → MVPA: 0.73 (0.57, 0.94) MVPA → LPA: 1.23 (0.96, 1.58) MVPA → SB: 1.36 (1.06, 1.75) Model 2 SB → LPA: 0.92 (0.89, 0.94) SB → MVPA: 0.79 (0.63, 0.99) MVPA → LPA: 1.15 (0.92, 1.44) MVPA → SB: 1.26 (1.01, 1.58) Model 3 SB → LPA: 0.91 (0.86, 0.96) SB → MVPA: 0.70 (0.57, 0.85) MVPA → LPA: 1.30 (1.06, 1.59) MVPA → SB: 1.43 (1.17, 1.74) Reallocating 30 or 60 min from SB to LPA or MVPA was associated with a reduction in mortality risk after 5 years of follow-up.
Lee [62]	Adults (*n* = 7006) from the National Health and Nutrition Examination Survey, USA; prospective cohort	SB, LPA, MPA, VPA – waist-worn accelerometers; sleep – not assessed	All-cause mortality	Mekary et al. [12]/1 min and a dose-response analysis	Age, sex, education level, income, BMI, binge drinking, smoking status, energy intake by 24-h dietary recall, self-reported general health condition, high blood pressure, high cholesterol, type 2 diabetes, history of heart attack, stroke, and cancer.	Reallocating 1 min of SB to LPA or MPA demonstrated a J-shaped association with mortality, with the turning point at about 400 min and 15 min per day, respectively. Reallocating time from SB to VPA decreased all-cause mortality in a linear manner.
Loprinzi et al. [65]	Adults (*n* = 5377) from the 2003–2006 National Health and Nutrition Examination Survey, USA; prospective cohort	SB, LPA, MVPA – waist-worn accelerometers; sleep – not assessed	Mortality	Mekary et al. [12]/30 min	Age, sex, race–ethnicity, BMI, smoking status and education	HR (95% CI) SB → LPA: 0.87 (0.80, 0.95) SB → MVPA: 0.19 (0.06, 0.60) LPA → SB: 1.14 (1.04, 1.24) LPA → MVPA: 0.22 (0.07, 0.69) MVPA → SB: 5.03 (1.64, 15.40) MVPA → LPA: 4.40 (1.42, 13.56)
Matthews et al. [66]	Less active (*n* = 69,606) and more active (*n* = 85,008) individuals from the NIH-AARP Diet and Health Study, USA; prospective cohort	Sleep, SB, exercise and non-exercise activities, LPA, MVPA – self report	All-cause and cardiovascular mortality	Mekary et al. [12]/60 min	Age, education, smoking history, sleep duration, overall health, BMI, overall sitting, each type of physical activity, and the sum of overall sitting and physical activity time	HR (95% CI) All-cause mortality Less active individuals SB → exercise: 0.58 (0.54, 0.63) SB → non-exercise: 0.70 (0.66, 0.74) SB → household chores: 0.80 (0.74, 0.86) SB → lawn and garden: 0.49 (0.43, 0.56) SB → daily walking: 0.66 (0.57, 0.78) SB → LPA: 0.81 (0.75, 0.88) SB → MVPA: 0.58 (0.54, 0.62) More active individuals SB → exercise: 0.91 (0.88, 0.94) SB → non-exercise: 1.00 (0.98, 1.02) SB → household chores: 1.02 (0.99, 1.05) SB → lawn and garden: 0.97 (0.93, 1.01) SB → daily walking: 0.99 (0.94, 1.05) SB → LPA: 1.04 (1.01, 1.08) SB → MVPA: 0.96 (0.94, 0.98) The time reallocations for cardiovascular mortality were weaker and non-significant among less active men for household chores, daily walking, and LPA compared to women. An increase in the risk was noted when reallocating SB with household and LPA in more active men. A significant inverse association for replacing SB with lawn and garden activities in more active men was noted (the same was not observed among women).
Matthews et al. [67]	Adults (*n* = 4840) from the 2003–2006 National Health and Nutrition Examination Survey, USA; prospective cohort	SB, LPA, MVPA – waist-worn accelerometers; sleep – not assessed	Mortality risk	Mekary et al. [12]/60 min	Age, sex, race-ethnicity, alcohol consumption, smoking status, BMI, self-reported diabetes, coronary artery disease, stroke, cancer, and mobility limitation.	HR (95% CI) Overall SB → LPA: 0.82 (0.73, 0.92) SB → MVPA: 0.58 (0.44, 0.77) Less active individuals SB → LPA: 0.80 (0.69, 0.92) SB → MVPA: 0.37 (0.26, 0.54) More active individuals SB → LPA: 1.29 (0.95, 1.74) SB → MVPA: 0.92 (0.60, 1.43)
Schmid et al. [71]	Adults (*n* = 3702) from the National Health and Nutrition Examination Survey 2003–2004 and 2005–2006 cycles, USA; prospective cohort	SB, LPA, MVPA – accelerometers (location is not presented); sleep – not assessed	All-cause mortality, CVD mortality, cancer mortality	Mekary et al. [12]/30 min	Age, sex, total accelerometer wear time (model 1). Model 2 is additionally adjusted for education, ethnicity, height, smoking, alcohol consumption, total dietary fat intake, total dietary fibre intake, mobility limitations, history of diabetes, history of coronary heart disease, history of congestive heart failure, history of stroke, history of cancer. Model 3 is the same as model 2 plus waist circumference.	HR (95% CI) All-Cause Mortality Model 1 SB → LPA: 0.85 (0.81, 0.88) SB → MVPA: 0.38 (0.22, 0.63) SB → LPA and MVPA: 0.82 (0.79, 0.85) LPA → MVPA: 0.45 (0.26, 0.75) Model 2 SB → LPA: 0.86 (0.83, 0.90) SB → MVPA: 0.50 (0.31, 0.80) SB → LPA and MVPA: 0.85 (0.81, 0.88) LPA → MVPA: 0.58 (0.36, 0.93) Model 3 SB → LPA: 0.88 (0.84, 0.92) SB → MVPA: 0.51 (0.32, 0.83) SB → LPA and MVPA: 0.86 (0.82, 0.90) LPA → MVPA: 0.58 (0.36, 0.95) CVD Mortality Model 1 SB → LPA: 0.84 (0.77, 0.90) SB → MVPA: 0.25 (0.09, 0.71) SB → LPA and MVPA: 0.80 (0.74, 0.86) LPA → MVPA: 0.30 (0.11, 0.86) Model 2 SB → LPA: 0.86 (0.79, 0.93) SB → MVPA: 0.35 (0.14, 0.92) SB → LPA and MVPA: 0.83 (0.77, 0.90) LPA → MVPA: 0.41 (0.16, 1.08) Model 3 SB → LPA: 0.88 (0.81, 0.95) SB → MVPA: 0.36 (0.13, 0.95) SB → LPA and MVPA: 0.85 (0.78, 0.92) LPA → MVPA: 0.41 (0.15, 1.11) Cancer mortality Model 1 SB → LPA: 0.91 (0.85, 0.98) SB → MVPA: 0.53 (0.22, 1.31) SB → LPA and MVPA: 0.88 (0.82, 0.96) LPA → MVPA: 0.58 (0.24, 1.44) Model 2 SB → LPA: 0.92 (0.85, 0.99) SB → MVPA: 0.69 (0.32, 1.50) SB → LPA and MVPA: 0.90 (0.83, 0.98) LPA → MVPA: 0.75 (0.34, 1.63) Model 3 SB → LPA: 0.93 (0.86, 1.01) SB → MVPA: 0.79 (0.39, 1.62) SB → LPA and MVPA: 0.92 (0.85, 1.00) LPA → MVPA: 0.85 (0.41, 1.73)
Stamatakis et al. [72]	Adults (*n* = 201,129) from the 45 and Up study from New South Wales, Australia; prospective cohort	Sleep, SB, LPA, MVPA – self report	All-cause mortality	Mekary et al. [12]/60 min	Age, sex, educational level, marital status, residence, BMI, smoking status, self-rated health, receiving help with daily tasks for a long-term illness or disability, prevalent disease at baseline, psychological distress, mutually adjusted for all activity classes, and total time in all activity classes.	HR (95% CI) Sleeping (≤7 h) → screen-time: 1.01 (0.98, 1.05) Sleeping (≤7 h) → sitting: 1.03 (0.99, 1.07) Sleeping (≤7 h) → standing: 0.98 (0.94, 1.02) Sleeping (≤7 h) → walking: 0.93 (0.84, 1.03) Sleeping (≤7 h) → MVPA: 0.90 (0.85, 0.96) Sleeping (≤7 h) → total activity: 1.01 (0.98, 1.04) Sleeping (> 7 h) → screen-time: 0.95 (0.93, 0.97) Sleeping (> 7 h) → sitting: 0.96 (0.94, 0.98) Sleeping (> 7 h) → standing: 0.92 (0.9, 0.94) Sleeping (> 7 h) → walking: 0.80 (0.75, 0.86) Sleeping (> 7 h) → MVPA: 0.84 (0.81, 0.87) Sleeping (> 7 h) → total activity: 1.06 (1.04, 1.07) Screen-time → sleeping (≤7 h): 0.95 (0.91, 0.99) Screen-time → sleeping (> 7 h): 1.06 (1.04, 1.09) Screen-time → sitting: 1.01 (1.00, 1.03) Screen-time → standing: 0.97 (0.95, 0.98) Screen-time → walking: 0.87 (0.82, 0.92) Screen-time → MVPA: 0.89 (0.86, 0.91) Screen-time → total activity: 1.01 (1.00, 1.02) Sitting → sleeping (≤7 h): 0.94 (0.90, 0.98) Sitting → sleeping (> 7 h): 1.05 (1.03, 1.07) Sitting → screen-time: 0.99 (0.97, 1.00) Sitting → standing: 0.95 (0.94, 0.96) Sitting → walking: 0.86 (0.81, 0.90) Sitting → MVPA: 0.88 (0.85, 0.90) Sitting → total activity: 1.02 (1.01, 1.03) Standing → sleeping (≤7 h): 0.99 (0.95, 1.03) Standing → sleeping (> 7 h): 1.10 (1.08, 1.13) Standing → screen-time: 1.04 (1.02, 1.05) Standing → sitting: 1.05 (1.04, 1.06) Standing → walking: 0.90 (0.85, 0.95) Standing → MVPA: 0.92 (0.89, 0.95) Standing → total activity: 0.98 (0.97, 0.99) Walking → sleeping (≤7 h): 1.10 (1.03, 1.18) Walking → sleeping (> 7 h): 1.17 (1.12, 1.21) Walking → screen-time: 1.15 (1.09, 1.22) Walking → sitting: 1.17 (1.11, 1.23) Walking → standing: 1.11 (1.05, 1.18) Walking → MVPA: 1.02 (0.96, 1.09) Walking → total activity: 0.88 (0.83, 0.93) MVPA → sleeping (≤7 h): 1.07 (1.02, 1.13) MVPA → sleeping (> 7 h): 1.18 (1.14, 1.22) MVPA → screen-time: 1.13 (1.09, 1.16) MVPA → sitting: 1.14 (1.11, 1.18) MVPA → standing: 1.09 (1.06, 1.12) MVPA → walking: 0.98 (0.92, 1.04) MVPA → total activity: 0.90 (0.87, 0.92)
Wijndaele et al. [39]	Middle-aged adults (*n* = 423,659) from the UK Biobank cohort study, UK; prospective cohort	SB (leisure screen time, TV viewing, computer usage) and leisure/home activities (walking for pleasure, light and heavy do-it-yourself), structured exercise, sleep – self reported	Mortality risk	Mekary et al. [12]/30 min	Townsend deprivation index, alcohol intake, smoking status, salt adding behaviour, oily fish consumption, fruit and vegetable intake, processed and sleep duration, chronic disease status, parental history of cardiovascular disease or diabetes	HR (95% CI) Screen time → leisure/home activities: 0.95 (0.94, 0.97) Screen time → structured exercise: 0.87 (0.84, 0.90) Screen time → walking for pleasure: 0.95 (0.92, 0.98) Screen time → light do-it-yourself: 0.97 (0.94, 1.00) Screen time → heavy do-it-yourself: 0.93 (0.90, 0.96) Screen time → strenuous sports: 0.87 (0.79, 0.95) Screen time → other exercises: 0.88 (084, 0.91) TV viewing → leisure/home activities: 0.94 (0.93, 0.96) TV viewing → structured exercise: 0.87 (0.84, 0.90) TV viewing → walking for pleasure: 0.94 (0.92, 0.97) TV viewing → light do-it-yourself: 0.96 (0.94, 0.99) TV viewing → heavy do-it-yourself: 0.91 (0.89, 0.95) TV viewing → strenuous sports: 0.86 (0.79, 0.95) TV viewing → other exercises: 0.87 (0.83, 0.91) Men Computer usage → leisure/home activities: 0.98 (0.96, 1.00) Computer usage → structured exercise: 0.89 (0.85, 0.93) Computer usage → walking for pleasure: 0.98 (0.95, 1.01) Computer usage → light do-it-yourself: 0.99 (0.96, 1.02) Computer usage → heavy do-it-yourself: 0.97 (0.93, 1.00) Computer usage → strenuous sports: 0.90 (0.81, 0.99) Computer usage → other exercises: 0.88 (0.84, 0.93) Women Computer usage → leisure/home activities: 0.94 (0.91, 0.97) Computer usage → structured exercise: 0.90 (0.84, 0.96) Computer usage → walking for pleasure: 0.93 (0.89, 0.98) Computer usage → light do-it-yourself: 0.99 (0.94, 1.04) Computer usage → heavy do-it-yourself: 0.84 (0.76, 0.94) Computer usage → strenuous sports: 0.88 (0.73, 1.06) Computer usage → other exercises: 0.90 (0.84, 0.97)

*SB* sedentary behaviour, *LPA* light intensity physical activity, *MVPA* moderate-to-vigorous intensity physical activity, *MPA* moderate intensity physical activity, *VPA* vigorous intensity physical activity, *CVD* cardiovascular disease, *BMI* body mass index, *MET* metabolic equivalent of task, *HR* hazard ratio, *CI* confidence interval

**Table 2:** Summary of the findings from the studies assessing perceived/general health status

Study	Sample and study design	Measures of sleep, SB, LPA, MVPA	Outcome measures	Data analysis method/reallocated time	Adjustments for confounding	Results
Balboa-Castillo et al. [45]	Older adults (*n* = 1097), Spain; prospective cohort	Sleep, SB, housework, leisure-time physical activity (LPA, MVPA) – self-report	HRQoL	Mekary et al. [12]/60 min	Age, sex, educational level, size of municipality of residence, consumption of tobacco, consumption of alcohol, self-reported diseases diagnosed by a physician, score on appropriate SF-36 scale in 2003, number of hours lying or sleeping, and total number of hours spent in all types of physical activity.	β (95% CI) Physical functioning SB ↔ LPA: 3.41 (0.81, 6.00) SB ↔ MVPA: 4.14 (1.92, 6.37) SB ↔ housework: 1.04 (0.25, 1.85) Physical role SB ↔ LPA: 10.61 (6.08, 15.13) SB ↔ MVPA: 1.19 (− 2.71, 5.10) SB ↔ housework: 1.68 (0.27, 3.01) Bodily pain SB ↔ LPA: 4.22 (1.19, 7.26) SB ↔ MVPA: 2.93 (0.31, 5.56) SB ↔ housework: 1.05 (0.10, 1.99) General health SB ↔ LPA: 2.44 (0.66, 4.23) SB ↔ MVPA: − 0.06 (− 1.61, 1.50) SB ↔ housework: 0.31 (− 0.24, 0.87) Vitality SB ↔ LPA: 4.14 (1.58, 6.71) SB ↔ MVPA: 2.51 (0.29, 4.73) SB ↔ housework: 0.67 (− 0.13, 1.47) Social functioning SB ↔ LPA: 4.80 (1.84, 7.77) SB ↔ MVPA: 2.06 (− 0.47, 4.61) SB ↔ housework: 1.08 (0.14, 2.00) Emotional role SB ↔ LPA: 4.93 (0.98, 8.87) SB ↔ MVPA: 1.03 (− 2.40, 4.46) SB ↔ housework: 1.21 (− 0.03, 2.45) Mental health SB ↔ LPA: 2.51 (0.17, 4.86) SB ↔ MVPA: 0.53 (− 1.51, 2.57) SB ↔ housework: 0.83 (0.09, 1.57)
Buman et al. [47]	Older adults (*n* = 862) from the 2005–2007 Senior Neighborhood Quality of Life Study, USA; prospective cohort	SB, MVPA (“high-light” physical activity and “low-light” physical activity) – waist-worn accelerometers; sleep – not assessed	Physical health and psychosocial well-being factors	Mekary et al. [12]/30 min	Age, sex, race, educational status, marital status, senior housing status, current smoking status and walkability index.	β (95% CI) Physical health SB → “low-light” physical activity: 0.07 (0.04, 0.09) SB → “high-light” physical activity: 0.30 (0.19, 0.42) SB → MVPA: 0.34 (0.23, 0.44) “Low-light” physical activity → SB: − 0.07 (− 0.09, − 0.04) “Low-light” physical activity → “high-light” physical activity: 0.24 (0.11, 0.36) “Low-light” physical activity → MVPA: 0.21 (0.10, 0.32) “High-light” physical activity → SB: − 0.30 (− 0.42, − 0.19) “High-light” physical activity → “low-light” physical activity: − 0.24 (− 0.36, − 0.11) “High-light” physical activity → MVPA: − 0.27 (− 0.55, 0.01) MVPA → SB: − 0.17 (− 0.22, − 0.12) MVPA → “low-light” physical activity: − 0.10 (− 0.16, − 0.05) MVPA → “high-light” physical activity: 0.14 (− 0.06, 0.27) Psychosocial well-being SB → “low-light” physical activity: 0.01 (− 0.02, 0.04) SB → “high-light” physical activity: 0.24 (0.12, 0.36) SB → MVPA: − 0.02 (− 0.13, 0.10) “Low-light” physical activity → SB: − 0.01 (− 0.04, 0.02) “Low-light” physical activity → “high-light” physical activity: 0.24 (0.10, 0.37) “Low-light” physical activity → MVPA: − 0.03 (− 0.15, 0.08) “High-light” physical activity → SB: − 0.24 (− 0.36, − 0.12) “High-light” physical activity → “low-light” physical activity: − 0.24 (− 0.37, − 0.10) “High-light” physical activity → MVPA: − 0.50 (− 0.81, − 0.19) MVPA → SB: 0.01 (− 0.05, 0.07) MVPA → “low-light” physical activity: 0.02 (− 0.04, 0.08) MVPA → “high-light” physical activity: 0.25 (0.10, 0.41)
Fanning et al. [26]	Low-active healthy older adults (*n* = 247), USA; cross-sectional	SB, LPA, MVPA – waist-worn accelerometers; sleep – self report	Self-regulation, spatial working memory, task-switching	Mekary et al. [12]/30 min	Age, sex, race.	β (standard error) Self-regulation – total score SB → LPA: 0.60 (0.38) SB → MVPA: 1.81 (0.80) SB → sleep: 1.29 (0.39) Self-regulation – self-monitoring SB → LPA: 0.15 (0.09) SB → MVPA: 0.27 (0.19) SB → sleep: 0.23 (0.09) Self-regulation – goal setting SB → LPA: 0.11 (0.08) SB → MVPA: 0.27 (0.18) SB → sleep: 0.32 (0.09) Self-regulation – social support SB → LPA: 0.09 (0.06) SB → MVPA: 0.08 (0.13) SB → sleep: 0.18 (0.07) Spatial working memory – item 2 reaction time SB → LPA: − 0.16 (6.20) SB → MVPA: − 4.46 (13.09) SB → sleep: 1.13 (6.50) Spatial working memory – item 3 reaction time SB → LPA: − 0.19 (6.18) SB → MVPA: − 5.60 (13.06) SB → sleep: − 1.11 (6.49) Spatial working memory – item 4 reaction time SB → LPA: − 5.87 (6.57) SB → MVPA: − 4.61 (13.88) SB → sleep: − 0.21 (6.89) Spatial working memory – item 2 accuracy SB → LPA: − 0.01 (0.00) SB → MVPA: 0.03 (0.01) SB → sleep: − 0.00 (0.01) Spatial working memory – item 3 accuracy SB → LPA: − 0.01 (0.01) SB → MVPA: 0.02 (0.01) SB → sleep: − 0.00 (0.01) Spatial working memory – item 4 accuracy SB → LPA: − 0.00 (0.01) SB → MVPA: 0.01 (0.01) SB → sleep: − 0.00 (0.01) Task-switching – single reaction time SB → LPA: 7.45 (5.01) SB → MVPA: − 23.12 (10.63) SB → sleep: 4.12 (5.31) Task-switching – mixed-repeat reaction time SB → LPA: 5.00 (6.24) SB → MVPA: − 27.06 (13.24) SB → sleep: − 12.2 (6.61) Task-switching – mixed-switch reaction time SB → LPA: 1.04 (8.23) SB → MVPA: − 28.24 (17.45) SB → sleep: − 17.21 (8.71) Task-switching – local switch cost reaction time SB → LPA: − 3.93 (5.98) SB → MVPA: − 0.40 (12.67) SB → sleep: − 5.19 (6.33) Task-switching – global switch cost reaction time SB → LPA: − 2.84 (6.39) SB → MVPA: − 1.54 (13.55) SB → sleep: − 16.86 (6.77) Task-switching – single accuracy SB → LPA: − 0.00 (0.00) SB → MVPA: 0.01 (0.01) SB → sleep: − 0.01 (0.00) Task-switching – mixed-repeat accuracy SB → LPA: − 0.01 (0.01) SB → MVPA: 0.01 (0.01) SB → sleep: − 0.01 (0.01) Task-switching – mixed-switch accuracy SB → LPA: − 0.01 (0.01) SB → MVPA: 0.01 (0.01) SB → sleep: − 0.01 (0.01) Task-switching – local switch accuracy SB → LPA: 0.00 (0.00) SB → MVPA: 0.00 (0.01) SB → sleep: − 0.00 (0.00) Task-switching – global switch accuracy SB → LPA: 0.00 (0.01) SB → MVPA: − 0.00 (0.01) SB → sleep: 0.01 (0.01)
Loprinzi et al. [65]	Adults (*n* = 5377) from the 2003–2006 National Health and Nutrition Examination Survey, USA; prospective cohort	SB, LPA, MVPA – waist-worn accelerometers; sleep – not assessed	HRQoL	Mekary et al. [12]/30 min	Age, sex, race–ethnicity, BMI, smoking status and education	β (95% CI) HRQoL SB → LPA: 0.92 (0.85, 1.01) SB → MVPA: 0.28 (0.13, 0.58) LPA → SB: 1.07 (0.98, 1.17) LPA → MVPA: 0.30 (0.14, 0.65) MVPA→ SB: 3.54 (1.70, 7.40) MVPA → LPA: 3.3 (1.54, 7.02)
Vallance et al. [34]	Non-Hodgkin lymphoma survivors (*n* = 149) from the Western Australian Cancer Registry, Australia; cross-sectional	SB bouts and non-bouts, standing, LPA, MVPA bouts and non-bouts – waist-worn accelerometers; sleep – self report	Fatigue and HRQoL	Mekary et al. [12]/30 min	Sex, non-Hodgkin lymphoma type and time since diagnosis, country of birth, highest level of education, working status, comorbidity and non-Hodgkin lymphoma treatment	β (95% CI) Fatigue Sleep ↔ SB bouts: 0.1 (− 1.0, 1.1) Sleep ↔ SB non-bouts: 0.6 (− 0.5, 1.8) Sleep ↔ LPA: 0.3 (− 0.8, 1.4) Sleep ↔ MVPA non-bouts: 1.2 (− 2.4, 4.8) Sleep ↔ MVPA bouts: 5.7 (1.8, 9.7) SB bouts ↔ SB non-bouts: 0.5 (− 0.3, 1.4) SB bouts ↔ LPA: 0.2 (− 0.5, 0.9) SB bouts ↔ MVPA non-bouts: 1.1 (− 2.3, 4.5) SB bouts ↔ MVPA bouts: 5.7 (1.6, 9.7) SB non-bouts ↔ LPA: − 0.3 (− 1.5, 0.8) SB non-bouts ↔ MVPA non-bouts: 0.6 (− 2.6, 3.9) SB non-bouts ↔ MVPA bouts: 5.1 (1.0, 9.3) LPA ↔ MVPA non-bouts: 0.9 (− 2.9, 4.8) LPA ↔ MVPA bouts: 5.5 (1.5, 9.5) MVPA non-bouts ↔ MVPA bouts: 4.5 (− 1.4, 10.5) HRQoL Sleep ↔ SB bouts: − 0.6 (− 1.9, 0.8) Sleep ↔ SB non-bouts: − 0.2 (− 1.7, 1.3) Sleep ↔ LPA: − 0.6 (− 2.1, 0.9) Sleep ↔ MVPA non-bouts: 0.0 (− 4.7, 4.8) Sleep ↔ MVPA bouts: 4.5 (− 0.8, 9.7) SB bouts ↔ SB non-bouts: 0.4 (− 0.8, 1.5) SB bouts ↔ LPA: − 0.1 (− 0.9, 0.8) SB bouts ↔ MVPA non-bouts: 0.6 (− 3.9, 5.1) SB bouts ↔ MVPA bouts: 5.0 (− 0.4, 10.4) SB non-bouts ↔ LPA: − 0.4 (− 1.9, 1.1) SB non-bouts ↔ MVPA non-bouts: 0.2 (− 4.1, 4.5) SB non-bouts ↔ MVPA bouts: 4.6 (− 0.8, 10.1) LPA ↔ MVPA non-bouts: 0.6 (− 4.4, 5.7) LPA ↔ MVPA bouts: 5.1 (− 0.2, 10.4) MVPA non-bouts ↔ MVPA bouts: 4.4 (− 3.5, 12.3)
Van Roekel et al. [73]	Colorectal cancer survivors (*n* = 145) from the Energy for life after ColoRectal cancer study, Netherlands; cross-sectional	Sleep, SB, standing, physical activity (defined as > 1.5 METs) – triaxial MOX activity monitor	HRQoL	Mekary et al. [12]/60 min	Age, sex, number of comorbidities, smoking status, time since diagnosis, cancer stage, BMI, perceived deficiency in social support score, chemotherapy received, stoma, tumor subsite, education level and having a partner.	β (95% CI) Global quality of life SB → standing: 1.0 (0.7, 1.5) SB → physical activity: 1.2 (0.6, 2.5) Standing → physical activity: 1.2 (0.5, 3.1) Physical functioning SB → standing: 1.1 (0.7, 1.7) SB → physical activity: 1.7 (0.8, 3.7) Standing → physical activity: 1.5 (0.5, 4.3) Role functioning SB → standing: 1.2 (0.8, 1.8) SB → physical activity: 0.7 (0.3, 1.5) Standing → physical activity: 0.6 (0.2, 1.6) Social functioning SB → standing: 1.2 (0.8, 1.8) SB → physical activity: 0.6 (0.3, 1.3) Standing → physical activity: 0.5 (0.2, 1.4) Disability SB → standing: 0.6 (0.4, 0.9) SB → physical activity: 0.9 (0.4, 1.9) Standing → physical activity: 1.6 (0.6, 4.2) Fatigue SB → standing: 1.0 (0.6, 1.4) SB → physical activity: 0.6 (0.3, 1.3) Standing → physical activity: 0.7 (0.3, 1.7) Depression SB → standing: 1.1 (0.7, 1.6) SB → physical activity: 0.8 (0.4, 1.6) Standing → physical activity: 0.8 (0.3, 1.9) Anxiety SB → standing: 1.1 (0.7, 1.5) SB → physical activity: 0.7 (0.4, 1.5) Standing → physical activity: 0.7 (0.3, 1.7)

*SB* sedentary behaviour, *LPA* light intensity physical activity, *MVPA* moderate-to-vigorous intensity physical activity, *MPA* moderate intensity physical activity, *HRQoL* health-related quality of life, *MET* metabolic equivalent of task, *CI* confidence interval

**Table 3:** Summary of the findings from the studies assessing mental health outcomes

Study	Sample and study design	Measures of sleep, SB, LPA, MVPA	Outcome measures	Data analysis method/reallocated time	Adjustments for confounding	Results
Janssen [60]	Children (*n* = 20,122) from the Canadian Health Behavior in School-aged Children study, Canada; cross-sectional	Sedentary video games, active outdoor play, active video games – self report; sleep – not assessed	Mental health outcomes	Mekary et al. [12]/60 min	Sex, school grade, household structure, race, immigration status, socioeconomic status, smoking, frequency of drunkenness, active travel to school, participation in sport, and a diet composition factor score.	PR (95% CI) High emotional problems Sedentary video games → active outdoor play: 0.94 (0.91, 0.97) Active outdoor play → active video games: 1.07 (1.03, 1.11) Active video games → sedentary video games: 1.07 (1.03, 1.10) Active video games → active outdoor play: 0.93 (0.90, 0.97) Sedentary video games → active outdoor play: 0.88 (0.85, 0.90) Active outdoor play → sedentary video games: 1.14 (1.11, 1.17) High life satisfaction Sedentary video games → active outdoor play: 1.04 (1.02, 1.07) Active outdoor play → active video games: 0.97 (0.95, 0.99) Active video games → sedentary video games: 0.96 (0.94, 0.98) Active video games → active outdoor play: 1.04 (1.01, 1.06) Sedentary video games → active outdoor play: 1.08 (1.06, 1.10) Active outdoor play → sedentary video games: 0.92 (0.91, 0.94) High prosocial behaviour Sedentary video games → active outdoor play: 1.13 (1.09, 1.16) Active outdoor play → active video games: 0.94(0.91, 0.98) Active video games → sedentary video games: 0.89 (0.86, 0.92) Active video games → active outdoor play: 1.06 (1.02, 1.10) Sedentary video games → active outdoor play: 1.19 (1.16, 1.22) Active outdoor play → sedentary video games: 0.84 (0.82, 0.86)
Mekary et al. [69]	Adult women (*n* = 32,900) from the Nurses’ Health Study, USA; prospective cohort	Television watching, easy walking pace, average walking pace, brisk/very brisk walking pace, jogging/running, other physical activities, total discretionary time – self report; sleep – not assessed	Risk of depression	Mekary et al. [12]/60 min	Age, weight, smoking, menopausal status, use of postmenopausal hormone therapy, previously diagnosed medical conditions, marital status, osteoarthritis, social or community group involvement, wealth and income, education, occupation, working status and educational level.	RR (95% CI) TV watching → easy walking: 1.32 (0.73, 2.38) TV watching → average walking: 0.88 (0.77, 1.00) TV watching → brisk walking: 0.76 (0.68, 0.85) TV watching → running: 0.76 (0.45, 1.29) TV watching → other activities: 0.93 (0.85, 1.01) TV watching → total discretionary time: 1.03 (1.02, 1.05) Easy walking → TV watching: 0.73 (0.42, 1.26) Easy walking → average walking: 0.65 (0.37, 1.12) Easy walking → brisk walking: 0.56 (0.32, 0.96) Easy walking → running: 0.56 (0.26, 1.19) Easy walking → other activities: 0.68 (0.39, 1.18) Easy walking → total discretionary time: 1.42 (0.82, 2.45) Average walking → TV watching: 1.13 (0.99, 1.29) Average walking → easy walking: 1.49 (0.82, 2.71) Average walking → brisk walking: 0.86 (0.74, 0.99) Average walking → running: 0.86 (0.50, 1.48) Average walking → other activities: 1.05 (0.89, 1.24) Average walking → total discretionary time: 0.91 (0.80, 1.04) Brisk walking → TV watching: 1.32 (1.18, 1.47) Brisk walking → easy walking: 1.73 (0.96, 3.13) Brisk walking → average walking: 1.16 (1.00, 1.34) Brisk walking → running: 1.00 (0.58, 1.72) Brisk walking → other activities: 1.22 (1.05, 1.42) Brisk walking → total activity: 0.79 (0.70, 0.88) Running → TV watching: 1.19 (0.74, 1.91) Running → easy walking: 1.56 (0.74, 3.32) Running → average walking: 1.05 (0.64, 1.70) Running → brisk walking: 0.90 (0.55, 1.46) Running → other activities: 1.10 (0.68, 1.80) Running → total discretionary time: 0.87 (0.54, 1.40) Other activities → TV Watching: 1.08 (0.99, 1.17) Other activities → easy walking: 1.42 (0.78, 2.56) Other activities → average walking: 0.95 (0.80, 1.11) Other activities → brisk walking: 0.81 (0.70, 0.95) Other activities → running: 0.82 (0.48, 1.40) Other activities → total discretionary time: 0.96 (0.88, 1.05)
Rethorst et al. [31]	Adults (*n* = 11,116) from the Hispanic Community Health Study/Study of Latinos, USA; cross-sectional	SB, LPA, MPA, VPA – waist-worn accelerometers; sleep – not assessed	Depressive symptoms	Mekary et al. [12]/60 min	Age, sex, Hispanic background group, BMI, household income level, and education level, recruitment site, physical health and acculturation.	β (95% CI) SB ↔ LPA: − 0.003 (− 0.113, 0.107) SB ↔ MPA: 0.285 (− 0.264, 0.834) SB ↔ VPA: − 1.215 (− 2.250, − 0.180) LPA ↔ MPA: 0.288 (− 0.304, 0.880) LPA ↔ VPA: − 1.212 (− 2.244, − 0.179) MPA ↔ VPA: − 1.5 (− 2.883, − 0.116)

*SB* sedentary behaviour, *LPA* light intensity physical activity, *MVPA* moderate-to-vigorous intensity physical activity, *MPA* moderate intensity physical activity, *VPA* vigorous physical activity, *BMI* body mass index, *PR* prevalence ratio, *RR* relative risk, *CI* confidence interval

**Table 4:** Summary of the findings from the studies assessing adiposity

Study	Sample	Measures of sleep, SB, LPA, MVPA	Outcome measures	Data analysis method / reallocated time	Adjustments for confounding	Results
Aggio et al. [44]	Children and youth (*n* = 353) from the Camden Active Spaces project, UK; cross-sectional	SB, LPA, MVPA – waist-worn accelerometers; sleep – not assessed	Body fat	Mekary et al. [12] / 60 min	Age, sex, ethnicity, height and school deprivation.	β (95% CI) Body fat SB ↔ LPA: − 0.774 (− 1.714, 0.167) SB ↔ MVPA: 4.187 (1.142, 7.233) LPA ↔ MVPA: − 4.961 (− 8.212, − 1.710)
Boyle et al. [19]	Adult breast cancer survivors (*n* = 256) from the ACCEL-Breast study, Australia; cross-sectional	SB (prolonged SB and non-prolonged SB), LPA, MVPA – waist-worn accelerometers; sleep – self report	Waist circumference, BMI	Mekary et al. [12] / 30 min	Age, socioeconomic status, comorbidity, and smoking status	β (95% CI) Waist circumference Sleep ↔ prolonged SB: 0.00 (− 0.85, 0.85) Sleep ↔ non-prolonged SB: − 0.94 (− 1.80, − 0.08) Sleep ↔ LPA: 0.20 (− 0.68, 1.09) Sleep ↔ MVPA: − 2.50 (− 4.45, − 0.56) Prolonged SB ↔ non-prolonged SB: − 0.94 (− 1.79, − 0.10) Prolonged SB ↔ LPA: 0.20 (− 0.34, 0.74) Prolonged SB ↔ MVPA: − 2.51 (− 4.38, − 0.64) Non-prolonged SB ↔ LPA: 1.14 (0.18, 2.10) Non-prolonged SB ↔ MVPA: − 1.56 (− 3.40, 0.27) LPA ↔ MVPA: − 2.71 (− 4.72, − 0.69) BMI Sleep ↔ prolonged SB: 0.17 (− 0.20, 0.55) Sleep ↔ non-prolonged SB: − 0.23 (− 0.62, 0.15) Sleep ↔ LPA: 0.10 (− 0.30, 0.49) Sleep ↔ MVPA: − 0.75 (− 1.61, 0.11) Prolonged SB ↔ non-prolonged SB: − 0.41 (− 0.79, − 0.03) Prolonged SB ↔ LPA: − 0.08 (− 0.32, 0.16) Prolonged SB ↔ MVPA: − 0.93 (− 1.75, − 0.10) Non-prolonged SB ↔ LPA: 0.33 (− 0.10, 0.77) Non-prolonged SB ↔ MVPA: − 0.52 (− 1.34, 0.30) LPA ↔ MVPA: − 0.85 (− 1.75, 0.05)
Buman et al. [48]	Adults (*n* = 2185 – full sample; *n* = 923 – fasting sub-sample) from the 2005–2006 US National Health and Nutrition Examination Survey, USA; cross-sectional	SB, LPA, MVPA – waist-worn accelerometers; sleep – self report	Waist circumference	Mekary et al. [12] / 30 min	Sex, race, marital status, education, work status, income, smoking, depressive symptoms, 24-h dietary recalls estimating intakes of total energy, saturated fat, caffeine, and alcohol, a general health rating, diagnosis of cancer, malignancy, CVD, or diabetes, and current use of diabetic, antihypertensive, lipidemic, or other CVD medication.	RR (95% CI) Waist circumference Sleep → MVPA: 0.976 (0.966, 0.985) SB → MVPA: 0.973 (0.965, 0.981) LPA → MVPA: 0.974 (0.966, 0.983) Sleep → LPA: 1.001 (0.998, 1.005) SB → LPA: 0.999 (0.996, 1.001) SB → sleep: 0.997 (0.993, 1.001)
Carson et al. [49]	Children and youth aged 6–17 years (*n* = 4169 – full sample; *n* = 1242 – fasting sub-sample) from the Canadian Health Measures Survey, Canada; cross-sectional	SB, LPA, MVPA – waist-worn accelerometers; sleep – self report	BMI, waist circumference, blood pressure, behavioural strengths and difficulties, CRF – full sample. Triglycerides, HDL, C-reactive protein, and insulin – fasting subsample.	Chastin et al. [13] / 10 min	Age, sex, and highest household education.	Reallocating 10 min of MVPA to SB, LPA or sleep, resulted in a 5.1, 1.2, and 1.1% increase in BMI z score, respectively. Reallocating 10 min of SB, LPA or sleep, to MVPA resulted in a less than 1% decrease in BMI z score. Similar effects were noted across all health indicators.
Chastin et al. [13]	Adults (*n* = 1937) from the National Health and Nutrition Examination Survey 2005–2006 cycle, USA; cross-sectional	SB, LPA, MVPA – waist-worn accelerometers; sleep – self report	BMI	Chastin et al. [13] / 10 min	Age, sex, ethnicity/race, self-reported health, diagnosis of health conditions, educational level, social economic status, smoking status, alcohol consumption, calorie intake, caffeine and fat, medications for diabetes and/or high blood pressure.	Reallocating 10 min of MVPA to SB resulted in a 1.2% change in BMI.
Collings et al. [20]	Children (*n* = 410) from the Physical Activity and Nutrition in Children study, Finland; cross-sectional	Sleep, SB, LPA, MPA, VPA – heart rate and movement sensor	BMI, body composition (FMI, TFMI, FFMI),	Mekary et al. [12] / 10 min	Age, sex, monitor wear characteristics, income, sleep duration, energy intake, frequency of breakfast consumption, number of meals per day, snacking, birth weight, maternal and paternal BMI. When FMI, TFMI and FFMI were outcomes further adjustment for CRF was made. CRF was adjusted for FMI.	β (95% CI) FMI SB → LPA: − 1.2 (− 1.8, − 0.64) SB → MPA: − 1.7 (− 2.7, − 0.78) SB → VPA: − 11.8 (− 17.4, − 5.8) LPA → MPA: − 0.51 (− 1.7, 0.73) LPA → VPA: − 10.7 (− 16.2, − 4.8) MPA → VPA: − 10.2 (− 16.4, − 3.5) TFMI SB → LPA: − 1.5 (− 2.1, − 0.82) SB → MPA: − 2.0 (− 3.2, − 0.83) SB → VPA: − 13.1 (− 20.1, − 5.5) LPA → MPA: − 0.58 (− 2.0, 0.86) LPA → VPA: − 11.8 (− 18.8, − 4.3) MPA → VPA: − 11.3 (− 19.0, − 2.9) FFMI SB → LPA: − 0.0026 (− 0.013, 0.0079) SB → MPA: 0.016 (− 0.0002, 0.031) SB → VPA: − 0.042 (− 0.12, 0.033) LPA → MPA: 0.018 (− 0.0042, 0.041) LPA → VPA: -0.039 (− 0.11, 0.034) MPA → VPA: − 0.057 (− 0.14, 0.024)
Collings et al. [21]	Children (*n* = 333) from the Born in Bradford birth cohort study, UK; cross-sectional	SB, LPA, MVPA – waist-worn accelerometers; sleep – self report or estimated from accelerometers	BMI, waist circumference, sum of skinfolds	Mekary et al. [12] / 20 min	Age, sex, ethnicity, index of multiple deprivation, monitor wear time, season of assessment and height.	β (95% CI) BMI SB → LPA: − 0.0096 (− 0.055, 0.036) SB → MVPA: 0.052 (− 0.039, 0.14) LPA → MVPA: 0.061 (− 0.054, 0.18) Waist circumference SB → LPA: − 0.034 (− 0.21, 0.15) SB → MVPA: − 0.11 (− 0.46, 0.24) LPA → MVPA: − 0.077 (− 0.53, 0.37) Sum of skinfolds SB → LPA: 0.048 (− 0.37, 0.47) SB → MVPA: − 0.77 (− 1.46, − 0.084) LPA → MVPA: − 0.82 (− 1.71, 0.062)
Dahl-Petersen et al. [22]	Adults (*n* = 1497) from the Inuit Health in Transition study (2005–2010), Greenland; cross-sectional	SB, LPA, MPA, VPA –combined accelerometer and heart rate monitor; sleep – not assessed	BMI, waist circumference, visceral fat and subcutaneous abdominal adipose tissue	Mekary et al. [12] / 60 min	Age, sex, smoking and fraction of weekend wear time (model 1) and BMI (model 2).	β (95% CI) Model 1 BMI SB → LPA: − 0.21 (− 0.37, − 0.04) SB → MPA: − 0.47 (− 0.81, − 0.14) LPA → MPA: − 0.27 (− 0.67, 0.14) LPA → VPA: − 1.68 (− 2.89, − 0.46) Waist circumference SB → LPA: 0.57 (− 1.00, − 1.13) SB → MPA: − 1.13 (− 2.01, − 0.26) LPA → MPA: − 0.56 (− 1.63, 0.50) LPA → VPA: − 6.05 (− 9.20, − 2.30) Visceral fat SB → LPA: − 0.04 (− 0.11, 0.04) SB → MPA: − 0.23 (− 0.39, − 0.08) LPA → MPA: − 20 (− 0.38, − 0.02) LPA → VPA: − 0.66 (− 1.21, − 0.11) Sub-cutaneous fat SB → LPA: − 0.08 (− 0.12, − 0.03) SB → MPA: − 0.05 (− 0,15, 0.04) LPA → MPA: 0.02 (− 0.09, 0.14) LPA → VPA: − 0.67 (− 1.01, − 0.34) Model 2 Waist circumference SB → LPA: − 0.007 (− 0.16, 0.14) SB → MPA: − 0.01 (− 0.32, 0.30) LPA → MPA: − 0.002(− 0.37, 0.37) LPA → VPA: − 1.9(− 3.0, − 0.80) Visceral fat SB → LPA: 0.03 (− 0.02, 0.09) SB → MPA: − 0.10 (− 0.21, 0.02) LPA → MPA: − 0.13 (− 0.27, 0.007) LPA → VPA: − 0.17 (− 0.58,0.25) Sub-cutaneous fat SB → LPA: − 0.03 (− 0.06, 0.005) SB → MPA: 0.04 (− 0.02, 0.10) LPA → MPA: 0.07 (− 0.01, 0.15) LPA → VPA: − 0.33 (− 0.56, − 0.10)
Dalene et al. [23]	Samples of 6-year-olds (girls, *n* = 495–512; boys, *n* = 475–494) 9 (girls, *n* = 1198–1219; boys, *n* = 1225-1253) and 15-year-olds (girls, *n* = 778–850; boys, *n* = 766–824) from the Physical Activity among Norwegian Children Study, Norway; cross-sectional	SB, LPA, MPA, VPA – waist-worn accelerometers; sleep – not assessed	BMI, waist circumference	Mekary et al. [12] / 10 min	Age at baseline, sex, follow-up time, BMI and waist circumference at baseline.	β (95% CI) Cross-sectional analyses BMI 6-year-old girls SB → LPA: 0.10 (0.04, 0.17) SB → MPA: − 0.18 (− 0.35, − 0.01) SB → VPA: − 0.21 (− 0.58, 0.16) 6-year-old boys SB → LPA: 0.08 (0.02, 0.15) SB → MPA: 0.03 (− 0.05, 0.12) SB → VPA: − 0.32 (− 0.71, 0.06) 9-year-olds SB → LPA: − 0.32 (− 0.71, 0.06) SB → MPA: − 0.08 (− 0.15, − 0.02) SB → VPA: − 0.83 (1.04, − 0.63) 15-year-olds SB → LPA: 0.03 (− 0.02, 0.07) SB → MPA: 0.06 (− 0.02, 0.15) SB → VPA: − 0.56 (− 0.87, − 0.25) Prospective analyses SB → LPA: 0.05 (− 0.00, 0.11) SB → MPA: − 0.05 (− 0.14, 0.04) SB → VPA: 0.16 (− 0.17, 0.49) Waist circumference 6-year-old girls SB → LPA: 0.29 (0.13, 0.45) SB → MPA: − 0.47 (− 0.85, − 0.10) SB → VPA: − 0.15 (− 1.20, 0.90) 6-year-old boys SB → LPA: 0.15 (− 0.02, 0.33) SB → MPA: 0.06 (− 0.16, 0.29) SB → VPA: − 0.79 (− 1.68, 0.10) 9-year-olds SB → LPA: 0.17 (0.10, 0.25) SB → MPA: − 0.32 (− 0.46, − 0.18) SB → VPA: − 1.79 (− 2.36, − 1.23) 15-year-olds SB → LPA: 0.17 (0.06, 0.28) SB → MPA: 0.02 (− 0.20, 0.24) SB → VPA: − 1.08 (− 1.94, − 0.21) Prospective analyses SB → LPA: 0.07 (− 0.08, 0.23) SB → MPA: − 0.09 (− 0.37, 0.20) SB → VPA: − 0.43 (− 1.29, 0.42)
Ekblom-Bak et al. [51]	Adults (*n* = 836) from the Swedish Cardio Pulmonary bioImage Study, Sweden; cross-sectional	SB, LPA, MPA, VPA – waist-worn accelerometers; sleep – not assessed	Waist circumference	Mekary et al. [12] / 1, 5, 10, 15, 20, 25, 30, 60, 90 and 120 min	Age, sex, education level, smoking habits, perceived psychological stress, energy intake and wear time.	OR (95% CI) 10-min reallocation High waist circumference SB → LPA: 0.97 (0.95, 0.98) SB → MPA: 0.94 (0.88, 0.99) SB → VPA: 0.62 (0.48, 0.79)
Ekblom-Bak et al. [52]	Adults (*n* = 654) from the Swedish Cardio Pulmonary bioImage Study, Sweden; cross-sectional	SB, LPA, MVPA – waist-worn accelerometers; sleep – not assessed	Waist circumference	Mekary et al. [12] / 1, 5, 10, 15, 20, 25, 30, 60, 90 and 120 min	Sex, age, education, smoking, perceived psychosocial stress.	RR (95% CI) 30 min reallocation Waist circumference (women < 88 and men < 102) SB → LPA: 0.982 (0.962, 1.003) SB → MVPA: 0.931 (0.878, 0.987) Waist circumference (women ≥88 and men ≥102) SB → LPA: 0.981 (0.954, 1.009) SB → MVPA: 0.880 (0.816, 0.950)
Fairclough et al. [25]	Children (*n* = 169) from the Active Schools Skelmersdale study, UK; cross-sectional	SB, LPA, MVPA – wrist-worn accelerometers; sleep – estimated from the ActiGraph raw accelerations	BMI, waist circumference	Dumuid et al. [14] / 15 min	IMD decile, age, sex, and BMI.	Reallocating 15 min from MVPA to sleep, SB or LPA predicted higher adiposity. Reallocating time to MVPA from sleep, SB or LPA increased the magnitude of estimated detriments for adiposity. Furthermore, the detriments were larger in magnitude than the estimated benefits of time reallocation from MVPA to sleep, SB or LPA.
Falconer et al. [53]	Adults with type 2 diabetes (*n* = 519) from the Early Activity in Diabetes study, UK; cross-sectional	SB, LPA, MVPA – waist-worn accelerometers; sleep – not assessed	BMI, waist circumference	Mekary et al. [12] / 30 min	Age, sex, ethnic group, IMD score, accelerometer wear time, relevant diabetes or lipid-lowering drugs.	β (95% CI) BMI SB bouts → SB nonbouts: − 0.60 (− 1.0, − 0.21) SB bouts → LPA: − 0.26 (− 0.47, − 0.05) SB bouts →MVPA: − 2.19 (− 2.89, − 1.49) SB nonbouts →LPA: − 0.01 (− 0.38, 0.36) SB nonbouts →MVPA: − 1.87 (− 2.59, − 1.14) LPA → MVPA: − 2.00 (− 2.74, − 1.26) Waist circumference SB bouts → SB nonbouts: − 1.16 (− 2.08, − 0.25) SB bouts →LPA: − 0.87 (− 1.35, − 0.39) SB bouts →MVPA: − 4.56 (− 6.19, − 2.93) SB nonbouts →LPA: − 0.44 (− 1.30, 0.41) SB nonbouts →MVPA: − 3.97 (− 5.65, − 2.28) LPA →MVPA: − 3.93 (− 5.65, − 2.21)
Gupta et al. [55]	Blue-Collar Workers (*n* = 692) from the PHysical ACTivity cohort, Denmark; cross-sectional	SB, standing time, MVPA – thigh-worn accelerometers; sleep – self report	BMI, waist circumference and body fat percentage	Mekary et al. [12] / 30 min	Age, sex, smoking status, alcohol intake, dietary patterns, and total measured time.	β (95% CI) Whole day Waist circumference Total SB →standing: − 0.50 (− 0.81, − 0.18) Total SB →walking: 0.40 (− 0.53, 1.33) Total SB →MVPA: − 4.21 (− 6.94, − 1.47) Long SB bouts →standing: − 0.86 (− 1.22, − 0.5) Long SB bouts →walking: 0.57 (− 0.35, 1.5) Long SB bouts →MVPA: − 3.93 (− 6.62, − 1.23) Long SB bouts →moderate SB bouts: − 0.36 (− 0.89, 0.16) Long SB bouts →brief SB bouts: − 2.60 (− 3.55, − 1.65) Body fat percentage Total SB →standing: − 0.29 (− 0.45, − 0.13) Total SB →walking: 0.19 (− 0.26, 0.65) Total SB →MVPA: − 2.70 (− 4.03, − 1.37) Long SB bouts →standing: − 0.46 (− 0.65, − 0.27) Long SB bouts →walking: 0.27 (− 0.19, 0.72) Long SB bouts →MVPA: − 2.38 (− 3.7, − 1.06) Long SB bouts →moderate SB bouts: − 0.10 (− 0.38, 0.17) Long SB bouts →brief SB bouts: −1.43 (− 1.92, − 0.94) BMI Total SB →standing: − 0.17 (− 0.28, − 0.06) Total SB → walking:0.17 (− 0.15, 0.48) Total SB →MVPA: − 1.37 (− 2.29, − 0.44) Long SB bouts →standing: − 0.31 (− 0.44, − 0.17) Long SB bouts →walking: 0.18(− 0.14, 0.5) Long SB bouts →MVPA: − 1.28(− 2.2, − 0.35) Long SB bouts →moderate SBbouts: − 0.16 (− 0.35, 0.03) Long SB bouts →brief SB bouts: −0.82 (− 1.17, − 0.48) Work time Waist circumference Total SB →standing:− 0.24(− 0.6, 0.11) Total SB → walking:0.55 (− 0.38, 1.48) Total SB →MVPA: − 3.43 (− 6.25, − 0.61) Long SB bouts →standing: − 0.59 (− 1.16, − 0.03) Long SB bouts →walking: 0.67(− 0.36, 1.7) Long SB bouts →MVPA: − 3.42 (− 6.3, − 0.55) Long SB bouts →moderate SB bouts:− 0.08 (− 0.81, 0.66) Long SB bouts →brief SB bouts: −2.40 (− 3.43, − 1.36) Body fat percentage Total SB → standing:− 0.14 (− 0.32, 0.04) Total SB → walking:0.27 (− 0.19, 0.74) Total SB →MVPA: − 1.91 (− 3.29, − 0.52) Long SB bouts →standing: − 0.17(− 0.48, 0.13) Long SB bouts →walking: 0.47(− 0.06, 0.99) Long SB bouts →MVPA: − 1.50 (− 2.9, − 0.11) Long SB bouts →moderate SB bouts:0.20 (− 0.19, 0.6) Long SB bouts →brief SB bouts: −1.22 (− 1.76, − 0.68) BMI Total SB →standing: − 0.08(− 0.2, 0.05) Total SB → walking:0.25 (− 0.07, 0.57) Total SB →MVPA: − 1.03 (− 1.97, − 0.09) Long SB bouts →standing: − 0.17(− 0.38, 0.04) Long SB bouts →walking: 0.28 (− 0.08, 0.64) Long SB bouts →MVPA: − 0.96(− 1.92, 0.00) Long SB bouts →moderate SB bouts:− 0.02 (− 0.29, 0.26) Long SB bouts →brief SB bouts: −0.72 (− 1.1, − 0.35) Non-work time Waist circumference Total SB → standing:− 0.82 (− 1.26, − 0.37) Total SB → walking:− 0.02 (− 1.01, 0.97) Total SB → MVPA: −4.00 (− 6.75, − 1.26) Long SB bouts →standing: − 0.94 (− 1.39, − 0.49) Long SB bouts →walking: 0.33(− 0.62, 1.28) Long SB bouts →MVPA: − 3.77(− 6.51, − 1.03) Long SB bouts →moderate SB bouts: − 0.42 (− 0.97, 0.14) Long SB bouts → brief SB bouts: − 2.74 (− 3.77, − 1.72) Body fat percentage Total SB → standing: − 0.42 (− 0.65, − 0.19) Total SB → walking: 0.00 (− 0.49, 0.49) Total SB → MVPA: − 2.18 (− 3.53, − 0.84) Long SB bouts → standing: − 0.55 (− 0.78, − 0.32) Long SB bouts → walking: 0.09 (− 0.38, 0.55) Long SB bouts → MVPA: − 1.88 (− 3.22, − 0.55) Long SB bouts → moderate SB bouts: − 0.18 (− 0.46, 0.1) Long SB bouts → brief SB bouts: − 1.60 (− 2.11, − 1.09) BMI Total SB → standing: − 0.24 (− 0.4, − 0.08) Total SB → walking: 0.08 (− 0.25, 0.41) Total SB → MVPA: − 1.20 (− 2.12, − 0.28) Long SB bouts → standing: − 0.32 (− 0.48, − 0.16) Long SB bouts → walking: 0.13 (− 0.19, 0.45) Long SB bouts → MVPA: − 1.10 (− 2.02, − 0.18) Long SB bouts → moderate SB bouts: − 0.16 (− 0.36, 0.03) Long SB bouts → brief SB bouts: − 0.87 (− 1.22, − 0.52)
Hamer et al. [56]	Adults (*n* = 445) from the Whitehall II epidemiological cohort, UK; cross-sectional	SB, LPA, MVPA – waist-worn accelerometers; sleep – not assessed	BMI	Mekary et al. [12] / 10 min	Age, sex, smoking, employment grade, and current statin use.	β (95% CI) BMI SB ↔ LPA: − 0.002 (− 0.059, 0.056) SB ↔ MVPA: − 0.39 (− 0.54, − 0.24) LPA ↔ MVPA: − 0.39 (− 0.55, − 0.22)
Healy et al. [58]	A general population-based sample (*n* = 698) from the 2011/12 Australian Diabetes, Obesity, and Lifestyle Study, Australia; cross-sectional	SB, standing, stepping – thigh-worn accelerometers; sleep – self report	BMI, waist circumference	Mekary et al. [12] / 120 min	Age, sex, contraceptive pill use, blood pressure tablets, cholesterol tablets, diabetes medication, ethnicity, occupation and employment status, income, fibre intake, energy intake, energy-adjusted fibre intake, alcohol intake, sodium intake, potassium intake, fruit and vegetable serves.	RR (95% CI) BMI Sitting → standing: 0.99 (0.97, 1.02) Sitting → stepping: 0.90 (0.86, 0.95) Standing → stepping: 0.91 (0.86, 0.96) β (95% CI) Waist circumference Sitting → standing: − 0.53 (− 3.08, 2.05) Sitting → stepping: − 7.48 (− 10.80, − 4.17) Standing → stepping: − 6.97 (− 11.05, − 2.89)
Healy et al. [57]	Individuals with diagnosed type 2 diabetes (*n* = 279) from the Living Well with Diabetes intervention, Australia; cross-sectional	SB (prolonged SB and non-prolonged SB), LPA, MVPA – waist-worn accelerometers; sleep – not assessed	Waist circumference, BMI	Mekary et al. [12] / 30 min	Age, sex, BMI, waist circumference, log HbA1c, insulin use, oral hypoglycaemic use, use of glucagon-like-peptide-1 agents, diabetes duration, income, education, weight loss aids in last 6 months, smoking status, CVD-related condition, musculoskeletal condition, depression and/or anxiety, employment, place of birth, Caucasian, energy intake, diet quality score.	β (95% CI) Waist circumference Prolonged SB → non-prolonged SB: − 0.69 (− 1.46, 0.08) Prolonged SB → LPA: − 0.77 (− 1.33, − 0.22) Prolonged SB → MVPA: 0.64 (− 1.96, 3.24) Non-prolonged SB → LPA: − 0.08 (− 0.93, 0.76) Non-prolonged SB → MVPA: 1.33 (− 1.31, 3.96) LPA → MVPA: 1.41 (− 1.37, 4.19) BMI Prolonged SB → non-prolonged SB: − 0.35 (− 0.70, − 0.01) Prolonged SB → LPA: − 0.36 (− 0.61, − 0.11) Prolonged SB → MVPA: 0.20 (− 0.93, 1.32) Non-prolonged SB → LPA: − 0.01 (− 0.38, 0.37) Non-prolonged SB → MVPA: 0.55 (− 0.58, 1.68) LPA → MVPA: 0.56 (− 0.64, 1.76)
Huang et al. [59]	Children (*n* = 672) from the Understanding Children’s Activity and Nutrition cohort study, China; prospective cohort	SB (screen time, academic-related activities, other sedentary behaviours) and sleep – self report; LPA and MVPA – waist-worn accelerometers	BMI	Mekary et al. [12] / 30 min	Age, sex, snacking habit of the child, parental education, parental BMI, and marital status.	β (95% CI) Screen time ↔ academic-related activities: − 0.00 (− 0.06, 0.06) Screen time ↔ other SBs: − 0.12 (− 0.20, − 0.04) Screen time ↔ sleep: − 0.03 (− 0.13, 0.06) Screen time ↔ LPA: − 0.05 (− 0.12, 0.02) Screen time ↔ MVPA: − 0.42 (− 0.59, − 0.24) Academic-related activities ↔ other SBs: − 0.13 (− 0.21, − 0.04) Academic-related activities ↔ sleep: − 0.04 (− 0.14, 0.06) Academic-related activities ↔ LPA: − 0.05 (− 0.12, 0.02) Academic-related activities ↔ MVPA: − 0.42 (− 0.60, − 0.24) Other SBs ↔ academic-related activities: 0.13 (0.04, 0.21) Other SBs ↔ sleep: 0.09 (− 0.03, 0.20) Other SBs ↔ LPA: 0.07 (− 0.02, 0.16) Other SBs ↔ MVPA: − 0.30 (− 0.49, − 0.10) Sleep ↔ academic-related activities: 0.04 (− 0.06, 0.14) Sleep ↔ other SBs: − 0.09 (− 0.20, 0.03) Sleep ↔ LPA: − 0.01 (− 0.12, 0.10) Sleep ↔ MVPA: − 0.38 (− 0.59, − 0.18) LPA ↔ academic-related activities: 0.05 (− 0.02, 0.12) LPA ↔ other SBs: − 0.07 (− 0.16. 0.02) LPA ↔ sleep: 0.01 (− 0.10 to 0.12) LPA ↔ MVPA: − 0.37 (− 0.57, − 0.16) MVPA ↔ academic-related activities: 0.42 (0.24, 0.60) MVPA ↔ other SBs: 0.30 (0.10, 0.49) MVPA ↔ sleep: 0.38 (0.18, 0.59) MVPA ↔ LPA: 0.37 (0.16, 0.57)
Leppänen et al. [63]	Four year old children (*n* = 307) from the MINISTOP trial, Sweden; cross-sectional	Sleep, SB, LPA, MPA, VPA – wrist-worn accelerometers	FFMI	Mekary et al. [12] / 5 min	Maternal BMI and educational attainment, paternal BMI and educational attainment, child’s age and sex at the measurement and awake wearing time of the ActiGraph. Waist circumference was adjusted for height.	β (95% CI) FFMI SB ↔ LPA: − 0.01 (− 0.04, 0.02) SB ↔ MPA: 0.01 (− 0.02, 0.04) SB ↔ VPA: 0.17 (0.04, 0.30) LPA ↔ MPA: 0.02 (− 0.03, 0.08) LPA ↔ VPA: 0.18 (0.05, 0.30) MPA ↔ VPA: 0.16 (0.01, 0.31)
Leppänen et al. [27]	Four year old children (*n* = 138) from the MINISTOP trial, Sweden; prospective cohort	Sleep, SB, LPA, MPA, VPA – wrist-worn accelerometers	BMI, FMI, FFMI, body fat percentage, waist circumference	Mekary et al. [12] / 5 min	Child’s age, sex at measurement, awake wearing time, models with SB or MPA as exposures were adjusted for VPA, while models with VPA or MVPA were adjusted for SB.	Reallocating 5 min from SB to LPA or MPA to VPA at baseline was associated with an increase in FFMI and BMI.
Loprinzi et al. [65]	Children (*n* = 1036) and adolescents (*n* = 1608) from the 2003–2006 National Health and Nutrition Examination Survey, USA; prospective cohort	SB, LPA, MVPA – waist-worn accelerometers; sleep – not assessed	BMI, waist circumference, triceps and subscapularis skinfold, sex specific android body fat percent, gynoid body fat percent, total body fat percent	Mekary et al. [12] / 60 min	Age, sex, race-ethnicity, energy intake, poverty-to-income ratio, cotinine, and accelerometer wear time.	β (95% CI) BMI SB ↔ LPA: − 0.18 (− 0.53, 0.16) SB ↔ MVPA: − 1.20 (− 1.60, − 0.79) LPA ↔ MVPA: − 1.01 (− 1.57, − 0.45) BMI Percentile SB ↔ LPA: 0.17 (− 2.60, 2.95) SB ↔ MVPA: − 7.33 (− 11.88, − 2.78) LPA ↔ MVPA: − 7.50 (− 12.53, − 2.47) Waist circumference SB ↔ LPA: − 0.55 (− 1.50, 0.38) SB ↔ MVPA: − 3.81 (− 5.09, − 2.54) LPA ↔ MVPA: − 3.25 (− 4.91, − 1.59) Triceps skinfold SB ↔ LPA: − 0.13 (− 0.70, 0.43) SB ↔ MVPA: − 2.54 (− 3.30, − 1.78) LPA ↔ MVPA: − 2.40 (− 3.31, − 1.49) Subscapularis skinfold SB ↔ LPA: − 0.36 (− 0.80, 0.08) SB ↔ MVPA: − 1.66 (− 2.37, − 0.95) LPA ↔ MVPA: − 1.30 (− 1.97, − 0.63) Android body fat percent SB ↔ LPA: − 0.28 (− 1.59, 1.02) SB ↔ MVPA: − 6.62 (− 8.80, − 4.45) LPA ↔ MVPA: − 6.34 (− 9.40, − 3.27) Gynoid body fat percent SB ↔ LPA: − 0.07 (− 0.87, 0.71) SB ↔ MVPA: − 4.40 (− 5.74, − 3.07 LPA ↔ MVPA: − 4.32 (− 6.11, − 2.54) Total body fat percent (standard error) SB ↔ LPA: − 0.27 (0.58) SB ↔ MVPA: − 4.62 (0.77) LPA ↔ MVPA: − 4.34 (0.89)
Mekary et al. [12]	Adult women (*n* = 4558) from the Nurses’ Health Study II, USA; prospective cohort	TV watching, easy walking, average walking, brisk walking, running, other activities, total activity – self report; sleep – not assessed	Weight change	Mekary et al. [12] / 30 min	Baseline age, weight, height, alcohol intake, sugar-sweetened beverage intake, energy-adjusted trans-fat intake, energy-adjusted fibre intake, oral contraceptive use, parity and antidepressant use.	β (95% CI) TV watching ↔ slow walking: − 1.02 (− 1.55, − 0.48) TV watching ↔ brisk walking: − 2.16 (− 2.64, − 1.68) TV watching ↔ running: − 3.73 (− 4.89, _2.57) TV watching ↔ other activities: − 1.73 (− 2.13, − 1.33) TV watching ↔ total activity: 0.47 (0.36, 0.59) Slow walking ↔ brisk walking: − 1.14 (− 1.75, − 0.53) Slow walking ↔ running: − 2.71 (− 3.97, − 1.45) Slow walking ↔ other activities: − 0.72 (− 1.39, − 0.04) Slow walking ↔ total activity: − 0.54 (− 1.07, − 0.02) Brisk walking ↔ running: − 1.57 (− 2.82, − 0.33) Brisk walking ↔ other activities: 0.43 (− 0.22, 1.07) Brisk walking ↔ total activity: − 1.69 (− 2.15, − 1.22) Running ↔ other activities: 2.00 (0.77, 3.22) Running ↔ total activity: − 3.26 (− 4.41, − 2.10) Other activities ↔ total activity: − 1.26 (− 1.65, − 0.87)
Mekary et al. [68]	Adult men (n = 10,500) from the Health Professionals Follow-Up Study, USA; prospective cohort	TV watching, MVPA (aerobic activity), weight training, other activities; sleep – self report	Waist circumference	Mekary et al. [12] / 20 min	Baseline age and waist circumference total average alcohol intake, sugar-sweetened beverage intake, percent energy of trans-fat, energy-adjusted fibres, energy-adjusted glycemic load, smoking, antidepressant Intake, percent energy of protein intake, sleep duration, slow walking in addition to TV watching, MVPA, weight training, and other activities.	β (95% CI) TV watching ↔ MVPA (aerobic activity): −0.42 (− 0.50, − 0.34) TV watching ↔ weight training: − 0.76 (− 1.02, − 0.50) TV watching ↔ other activities: − 0.24 (− 0.37, − 0.11) TV watching ↔ total discretionary time: 0.08 (0.05, 0.12) MVPA (aerobic activity) ↔ weight training: − 0.34 (− 0.62, − 0.07) MVPA (aerobic activity) ↔ other activities: 0.17 (0.03, 0.31) MVPA (aerobic activity) ↔ total discretionary time: − 0.33 (− 0.39, − 0.26) Weight training ↔ other activities: 0.52 (0.23, 0.80) Weight training ↔ total discretionary time: − 0.67 (− 0.93, − 0.41) Other activities ↔ total discretionary time: − 0.15 (− 0.28, − 0.03)
Moore et al. [28]	Youth (*n* = 11,588) from the International Children’s Accelerometry Database, Brazil, Europe, and USA; cross-sectional	SB, LPA, MPA, VPA – waist-worn accelerometers; sleep – not assessed	Waist circumference	Mekary et al. [12] / not presented	Accelerometer cut points.	Reallocating LPA with VPA was associated with a 0.67 to 7.30 cm smaller waist circumference at the 50th to 90th percentiles
Nilsson et al. [29]	Older woman (*n* = 113) recruited from an newspaper ad, Sweden; cross-sectional	SB (accumulated, continuous), LPA, MVPA – waist-worn accelerometers; sleep – not assessed	Waist circumference	Mekary et al. [12] / 10 min	Medical history, self-rated health status, total energy intake, fat intake, and alcohol consumption.	β (95% CI) Waist circumference MVPA → LPA: 2.19 (1.45, 2.93) MVPA → accumulated SB: 1.78 (1.04, 2.57) MVPA → continuous SB: 2.08 (1.35, 2.8)
Rosique-Esteban et al. [32]	Adults (*n* = 5776) from the PREDIMED-PLUS trial, Spain; cross-sectional	Sleep, SB, LPA, MVPA – self report	Prevalence of obesity, abdominal obesity	Mekary et al. [12] / 60 min	Age, sex, education, marital and employment status, smoking habits, personal and family history of illness, medical conditions, medication use, and adherence to an energy-restricted Mediterranean diet.	RR (95% CI) Obesity MVPA → sleep: 0.95 (0.93, 0.97) MVPA → TV-viewing: 0.92 (0.90, 0.94) MVPA → LPA: 0.96 (0.93, 0.99) LPA → sleep: 0.98 (0.95, 1.01) LPA → TV-viewing: 0.95 (0.92, 0.98) Sleep → TV-viewing: 0.97 (0.96, 0.98) Abdominal obesity MVPA → sleep: 0.97 (0.96, 0.98) MVPA → TV-viewing: 0.97 (0.96, 0.98) MVPA → LPA: 0.97 (0.96, 0.98) LPA → sleep: 1.01 (0.99, 1.03) LPA → TV-viewing: 0.99 (0.98, 1.00) Sleep → TV-viewing: 0.99 (0.98, 1.00)
Sardinha et al. [70]	Children (*n* = 386) from the Lisbon Metropolitan area, Portugal; cross-sectional	SB, LPA, MVPA – waist-worn accelerometers; sleep – not assessed	BMI, waist circumference, TFMI, total body fat mass	Mekary et al. [12] / 15, 30 min	Age, sex, accelerometer wear time and in the prospective analysis results were further adjusted for baseline body composition.	β (95% CI) 30 min reallocation Cross-sectional analysis BMI SB → LPA: 0.03 (− 0.09, 0.12) SB → MVPA: − 0.21 (− 0.39, − 0.03) Waist circumference SB → LPA: 0.21 (− 0.81, 1.23) SB → MVPA: − 1.32 (− 3.06, 0.42) TFMI SB → LPA: − 0.09 (− 0.36, 0.15) SB → MVPA: − 0.81 (− 12.60, − 0.36) Total body fat mass SB → LPA: − 0.27 (− 0.78, 0.27) SB → MVPA: -1.62 (− 2.52, − 0.69) Prospective analyses BMI SB → LPA: 0.03 (− 0.06, 0.09) SB → MVPA: − 0.06 (− 0.18, 0.06) Waist circumference SB → LPA: − 0.21 (− 0.87, 0.45) SB → MVPA: − 1.11 (− 2.16, − 0.06) TFMI SB → LPA: 0.03 (− 0.09, 0.15) SB → MVPA: − 0.21 (− 0.39, 0.00) Total body fat mass SB → LPA: 0.09 (− 0.15, 0.33) SB → MVPA: − 0.48 (− 0.87, − 0.06) 15 min reallocation Cross-sectional analysis BMI SB → LPA: 0.02 (− 0.05, 0.06) SB → MVPA: − 0.11 (− 0.20, − 0.02) Waist circumference SB → LPA: 0.11 (− 0.41, 0.62) SB → MVPA: − 0.66 (− 1.53, 0.21) TFMI SB → LPA: − 0.05 (− 0.18, 0.08) SB → MVPA: − 0.41 (− 6.30, − 0.18) Total body fat mass SB → LPA: − 0.14 (− 0.39, 0.14) SB → MVPA: − 0.81 (− 1.26, − 0.35) Prospective analyses BMI SB → LPA: 0.02 (− 0.03, 0.05) SB → MVPA: − 0.03 (− 0.09, 0.03) Waist circumference SB → LPA: − 0.11 (− 0.44, 0.23) SB → MVPA: − 0.56 (− 1.08, − 0.03) TFMI SB → LPA: 0.02 (− 0.05, 0.08) SB → MVPA: − 0.11 (− 0.20, 0.00) Total body fat mass SB → LPA: 0.05 (− 0.08, 0.17) SB → MVPA: − 0.24 (− 0.44, − 0.03)
Van der Berg et al. [35]	Adults (*n* = 2213) from The Maastricht Study, Netherlands; cross-sectional	SB, standing, stepping – thigh-worn accelerometers; sleep – not assessed	Waist circumference, BMI	Mekary et al. [12] / 30 min	Age, sex, level of education, smoking status, alcohol consumption, energy intake, mobility limitation, and prevalent cardiovascular disease	OR (95% CI) Waist circumference SB → standing: − 0.405 (− 0.60, − 0.21) SB → stepping: − 1.422 (− 1.78, − 1.06) Standing → stepping: − 1.017 (− 1.47, − 0.56) BMI SB → standing: − 0.038 (− 0.11, 0.03) SB → stepping: − 0.480 (− 0.62, − 0.35) Standing → stepping: − 0.443 (− 0.61, − 0.27)
Varela-Mato et al. [37]	Adult male heavy goods vehicle drivers (n = 87), from a transport company from the East Midlands, UK; cross-sectional	SB, standing and stepping (LPA, MVPA) – thigh-worn accelerometers; sleep – estimated from the accelerometers and matched with the participants’ daily log	Waist circumference, BMI	Mekary et al. [12] / 30 min	Age, ethnicity, education levels, shift pattern, smoking, alcohol intake, fruit and vegetable consumption and BMI.	β (95% CI) Workdays Waist Circumference SB → standing: − 0.1 (− 1.4, 1.2) SB → LPA: − 0.6 (− 3.9, 2.7) SB → MVPA: − 6.5 (− 11.0, − 1.9) SB → sleep: 0.1 (− 0.3, 0.5) BMI SB → standing: 0.07 (− 0.4, 0.6) SB → LPA: − 0.7 (− 1.9, 0.5) SB → MVPA: − 1.5 (− 3.2, 0.2) SB → sleep: − 0.0 (− 0.2, 0.1) Non-workdays Waist Circumference SB → standing: − 0.4 (− 1.2, 2.1) SB → LPA: 0.4 (− 1.3, 2.1) SB → MVPA: − 0.8 (− 4.8, 3.2) SB → sleep: − 0.4 (− 1.1, 0.5) BMI SB → standing: 0.1 (− 0.5, 0.2) SB → LPA: − 0.2 (− 0.8, 0.4) SB → MVPA: 0.2 (− 1.2, 1.7) SB → sleep: − 0.3 (− 0.5,–0.05)

*SB* sedentary behaviour, *LPA* light intensity physical activity, *MVPA* moderate-to-vigorous intensity physical activity, *MPA* moderate intensity physical activity, *VPA* vigorous intensity physical activity; *BMI* body mass index, *HDL* high-density lipoprotein, *CVD* cardiovascular disease*, FFM* fat-free mass, *TFMI* trunk fat mass index, *FFMI* fat-free mass index, *FMI* fat mass index, *CRF* cardiorespiratory fitness, *IMD* indices of multiple deprivation, *CI* confidence interval, *RR* relative risk, *OR* odds ratio

**Table 5:** Summary of the findings from the studies assessing fitness outcomes

Study	Sample and study design	Measures of sleep, SB, LPA, MVPA	Outcome measures	Data analysis method / reallocated time	Adjustments for confounding	Results
Aggio et al. [44]	Children and youth (*n* = 353) from the Camden Active Spaces project, UK; cross-sectional	SB, LPA, MVPA – waist-worn accelerometers; sleep – not assessed	Hand grip strength, horizontal jump distance, flexibility, peak expiratory flow	Mekary et al. [12] / 60 min	Age, sex, ethnicity, height and school deprivation.	β (95% CI) Hand grip strength SB ↔ LPA: 0.509 (0.000, 1.019) SB ↔ MVPA: 0.511 (− 1.139, 2.161) LPA ↔ MVPA: 0.002 (− 1.760, 1.763) Horizontal jump distance SB ↔ LPA: 0.409 (− 2.252, 3.070) SB ↔ MVPA: 16.093 (7.476, 24.710) LPA ↔ MVPA: 15.684 (6.484, 24.885) Peak expiratory flow SB ↔ LPA: − 0.149 (− 7.569, 7.298) SB ↔ MVPA: 5.389 (− 18.728, 29.506) LPA ↔ MVPA: 5.538 (− 20.210, 31.287) Flexibility SB ↔ LPA: − 0.048 (− 0.935, 0.839) SB ↔ MVPA: 4.783 (1.910, 7.656) LPA ↔ MVPA: 4.831 (1.764, 7.899)
Collings et al. [20]	Children (*n* = 410) from the Physical Activity and Nutrition in Children study, Finland; cross-sectional	Sleep, SB, LPA, MPA, VPA – heart rate and movement sensor	CRF	Mekary et al. [12] / 10 min	Age, sex, monitor wear characteristics, income, sleep duration, energy intake, frequency of breakfast consumption, number of meals per day, snacking, birth weight, maternal and paternal BMI. When FMI, TFMI and FFMI were outcomes further adjustment for CRF was made. CRF was adjusted for FMI.	β (95% CI) SB → LPA: − 0.0038 (− 0.010, 0.0027) SB → MPA: 0.014 (0.0064, 0.022) SB → VPA: 0.098 (0.040, 0.16) LPA → MPA: 0.018 (0.0054, 0.031) LPA → VPA: 0.10 (0.046, 0.16) MPA → VPA: 0.083 (0.024, 0.14)
Ekblom-Bak et al. [52]	Adults (*n* = 654) from the Swedish Cardio Pulmonary bioImage Study, Sweden; cross-sectional	SB, LPA, MVPA – waist-worn accelerometers; sleep – not assessed	VO2max	Mekary et al. [12] / 1, 5, 10, 15, 20, 25, 30, 60, 90 and 120 min	Sex, age, education, smoking, perceived psychosocial stress.	OR (95% CI) 30 min reallocation VO2max (women < 32 and men < 35 ml·min-1·kg-1) SB → LPA: 0.953 (0.926, 0.982) SB → MVPA: 0.870 (0.794, 0.953) VO2max (women ≥32 and men ≥35 ml·min-1·kg-1) SB → LPA: 0.989 (0.966, 1.013) SB → MVPA: 0.904 (0.851, 0.960)
Fairclough et al. [25]	Children (*n* = 169) from the Active Schools Skelmersdale study, UK; cross-sectional	SB, LPA, MVPA – wrist-worn accelerometers; sleep – estimated from the ActiGraph raw accelerations	CRF	Dumuid et al. [14] / 15 min	IMD decile, age, sex, and BMI.	Reallocating 15 min from MVPA to sleep, SB or LPA predicted higher adiposity and lower CRF. Reallocating time to MVPA from sleep, SB or LPA increased the magnitude of estimated detriments for fitness and adiposity. Furthermore, the detriments were larger in magnitude than the estimated benefits of time reallocation from MVPA to sleep, SB or LPA.
Kim [61]	Older woman (*n* = 101), from the Itabashi ward, metropolitan Tokyo, Japan; cross-sectional	SB (prolonged SB and non-prolonged SB), LPA, MVPA – wrist-worn accelerometers; sleep – not assessed	Usual gait speed, maximum gait speed, 5-chair sit-to-stand, and timed up-and-go tests	Mekary et al. [12] / 30 min	Age, BMI, education, living conditions, smoking, alcohol consumption, number of medical conditions, and Tokyo metropolitan institute of gerontology score.	β (95% CI) Usual walking speed Prolonged SB → non-prolonged SB: 0.013 (− 0.017, 0.043) Prolonged SB → LPA: − 0.032 (− 0.076, 0.013) Prolonged SB → MVPA: 0.240 (0.133, 0.346) Non-prolonged SB → LPA: − 0.045 (− 0.090, 0.000) Non-prolonged SB → MVPA: 0.226 (0.120, 0.332) LPA → MVPA: 0.271 (0.137, 0.405) Maximum walking speed Prolonged SB → non-prolonged SB: − 0.010 (− 0.049, 0.030) Prolonged SB → LPA: − 0.052 (− 0.108, 0.004) Prolonged SB → MVPA: 0.240 (0.0105, 0.375) Non-prolonged SB → LPA: − 0.043 (− 0.099, 0.013) Non-prolonged SB → MVPA: 0.250 (0.116, 0.383) LPA → MVPA: 0.292 (0.124, 0.460) 5 chair sit-to-stand Prolonged SB → non-prolonged SB: 0.298 (− 0.031, 0626) Prolonged SB → LPA: − 0.024 (− 0.496, 0.447) Prolonged SB → MVPA: − 0.960 (− 2.130, 0.209) Non-prolonged SB → LPA: − 0.322 (− 0.802, 0.158) Non-prolonged SB → MVPA: − 1.258 (− 2.417, − 0.098) LPA → MVPA: − 0.937 (− 2.397, 0.522) Timed Up and Go Prolonged SB → non-prolonged SB: − 0.468 (− 0.824, − 0.112) Prolonged SB → LPA: − 0.065 (− 0.591, 0.461) Prolonged SB → MVPA: − 2.264 (− 3.557, − 0.970) Non-prolonged SB → LPA: 0.404 (− 0.128, 0.935) Non-prolonged SB → MVPA: − 1.796 (− 3.082, − 0.510) LPA → MVPA: − 2.199 (− 3.816, − 0.581)
Leppänen et al. [63]	Four year old children (*n* = 307) from the MINISTOP trial, Sweden; cross-sectional	Sleep, SB, LPA, MPA, VPA – wrist-worn accelerometers	20-m shuttle run test, handgrip strength, standing long jump test and a 4 × 10-m shuttle run test	Mekary et al. [12] / 5 min	Maternal BMI and educational attainment, paternal BMI and educational attainment, child’s age and sex at the measurement and awake wearing time of the ActiGraph.	β (95% CI) 20-m shuttle run test SB ↔ LPA: − 0.05 (− 0.12, 0.03) SB ↔ MPA: 0.01 (− 0.08, 0.10) SB ↔ VPA: 0.87 (0.53, 1.22) LPA ↔ MPA: 0.06 (− 0.08, 0.20) LPA ↔ VPA: 0.92 (0.59, 1.25) MPA ↔ VPA: 0.86 (0.47, 1.26) Handgrip strength SB ↔ LPA: − 0.00 (− 0.05, 0.04) SB ↔ MPA: 0.03 (− 0.02, 0.09) SB ↔ VPA: 0.17 (− 0.04, 0.38) LPA ↔ MPA: 0.04 (− 0.05, 0.12) LPA ↔ VPA: 0.17 (− 0.03, 0.38) MPA ↔ VPA: 0.14 (− 0.10, 0.38) Standing long jump SB ↔ LPA: − 0.46 (− 0.90, − 0.02) SB ↔ MPA: 0.40 (− 0.14, 0.94) SB ↔ VPA: 2.09 (0.01, 4.17) LPA ↔ MPA: 0.86 (0.00, 1.71) LPA ↔ VPA: 2.55 (0.53, 4.56) MPA ↔ VPA: 1.68 (− 0.74, 4.10) 4 × 10 m-shuttle run SB ↔ LPA: 0.03 (− 0.03, 0.08) SB ↔ MPA: − 0.00 (− 0.07, 0.07) SB ↔ VPA: − 0.62 (− 0.88, − 0.36) LPA ↔ MPA: − 0.03 (− 0.13, 0.08) LPA ↔ VPA: − 0.65 (− 0.90, − 0.40) MPA ↔ VPA: − 0.62 (− 0.92, − 0.32)
Leppänen et al. [27]	Four year old children (*n* = 138) from the MINISTOP trial, Sweden; prospective cohort	Sleep, SB, LPA, MPA, VPA – wrist-worn accelerometers	20-m shuttle run test, handgrip strength, standing long jump test and a 4 × 10-m shuttle run test	Mekary et al. [12] / 5 min	Child’s age, sex at measurement, awake wearing time, models with SB or MPA as exposures were adjusted for VPA, while models with VPA or MVPA were adjusted for SB.	Reallocating 5 min from SB to LPA or MPA to VPA at baseline was associated with a better handgrip strength and with longer jumps at 12-month follow-up.
Van der Velde et al. [36]	Adults (*n* = 2024) from The Maastricht Study, Netherlands; cross-sectional	SB, standing, LPA, MVPA – thigh-worn accelerometers; sleep – not assessed	CRF	Mekary et al. [12] / 60 min	Age, education level, type 2 diabetes, BMI, alcohol use, smoking status, cardio vascular disease, beta-blocker use, energy intake and mobility limitations	β (95% CI) Men SB → standing: 0.01 (− 0.02, 0.04) SB → LPA: 0.08 (0.03, 0.14) SB → MVPA: 0.49 (0.39, 0.59) Women SB → standing: − 0.00 (− 0.02, 0.02) SB → LPA: 0.10 (0.05, 0.16) SB → MVPA: 0.28 (0.19, 0.36)

*SB* sedentary behaviour, *LPA* light intensity physical activity, *MVPA* moderate-to-vigorous intensity physical activity, *MPA* moderate intensity physical activity, *VPA* vigorous intensity physical activity, *BMI* body mass index, *CRF* cardiorespiratory fitness, *VO2max* maximal oxygen consumption, *IMD* indices of multiple deprivation, *CI* confidence interval, *OR* odds ratio

**Table 6:** Summary of the findings from the studies assessing biomarkers

Study	Sample	Measures of sleep, SB, LPA, MVPA	Outcome measures	Data analysis method / reallocated time	Adjustments for confounding	Results
Buman et al. [48]	Adults (*n* = 2185 – full sample; *n* = 923 – fasting sub-sample) from the 2005–2006 US National Health and Nutrition Examination Survey, USA; cross-sectional	SB, LPA, MVPA – waist-worn accelerometers; sleep – self report	Systolic and diastolic blood pressure, HDL, C-reactive protein, LDL, plasma glucose, insulin, triglycerides, HOMA-S, HOMA-β	Mekary et al. [12] / 30 min	Sex, race, marital status, education, work status, income, smoking, depressive symptoms, 24-h dietary recalls estimating intakes of total energy, saturated fat, caffeine, and alcohol, a general health rating, diagnosis of cancer, malignancy, CVD, or diabetes, and current use of diabetic, antihypertensive, lipidemic, or other CVD medication.	RR (95% CI) HDL cholesterol Sleep → MVPA: 1.044 (1.019, 1.070) SB → MVPA: 1.046 (1.028, 1.065) LPA → MVPA: 1.043 (1.023, 1.064) Sleep → LPA: 1.001 (0.994, 1.008) SB → LPA: 1.003 (0.998, 1.008) SB → sleep: 1.002 (0.994, 1.011) Triglycerides Sleep → MVPA: 0.915 (0.851, 0.983) SB → MVPA: 0.914 (0.855, 0.977) LPA → MVPA: 0.931 (0.869, 0.998) Sleep → LPA: 0.983 (0.964, 1.002) SB → LPA: 0.981 (0.972, 0.991) SB → sleep: 0.999 (0.982, 1.016) Insulin Sleep → MVPA: 0.893 (0.803, 0.994) SB → MVPA: 0.874 (0.786, 0.970) LPA → MVPA: 0.895 (0.801, 1.000) Sleep → LPA: 0.998 (0.969, 1.029) SB → LPA: 0.976 (0.962, 0.991) SB → sleep: 0.978 (0.957, 1.000)
Carson et al. [49]	Children and youth aged 6–17 years (*n* = 4169 – full sample; *n* = 1242 – fasting sub-sample) from the Canadian Health Measures Survey, Canada; cross-sectional	SB, LPA, MVPA – waist-worn accelerometers; sleep – self report	BMI, waist circumference, blood pressure, behavioural strengths and difficulties, CRF – full sample. Triglycerides, HDL, C-reactive protein, and insulin – fasting subsample.	Chastin et al. [13] / 10 min	Age, sex, and highest household education.	Reallocating 10 min of MVPA to SB, LPA or sleep, resulted in a 5.1, 1.2, and 1.1% increase in BMI z score, respectively. Reallocating 10 min of SB, LPA or sleep, to MVPA resulted in a less than 1% decrease in BMI z score. Similar effects were noted across all health indicators.
Chastin et al. [13]	Adults (*n* = 1937) from the National Health and Nutrition Examination Survey 2005–2006 cycle, USA; cross-sectional	SB, LPA, MVPA – waist-worn accelerometers; sleep – self report	Blood pressure, plasma glucose and insulin, HDL, C-reactive protein, LDL, triglycerides	Mekary et al. [12] / 10 min	Age, sex, ethnicity/race, self-reported health, diagnosis of health conditions, educational level, social economic status, smoking status, alcohol consumption, calorie intake, caffeine and fat, medications for diabetes and/or high blood pressure.	Reallocating 10 min of LPA or MVPA to sleep was associated with positive effects on systolic and diastolic blood pressure. Reallocating 10 min of sleep to MVPA was associated with positive effects on HDL. Reallocating 10 min of MVPA with sleep was associated with positive effects on C-reactive protein. Reallocating 10 min of MVPA with sleep or SB was associated with detrimental effects on LDL, triglycerides, glucose and insulin level and HOMA. Similar, but smaller effects were noted when 10 min of SB was reallocated to LPA or sleep. Reallocating 10 min of MVPA to sleep or LPA had a detrimental effect on both obesity markers, with stronger magnitude when LPA replaced MVPA. Reallocating 10 min of SB or sleep to LPA was associated with more favourable effects on LDL, triglycerides, glucose, insulin, and HOMA. The effects were more pronounced when 10 min of LPA replaced sleep than SB.
Edwardson et al. [24]	Adults identified as being at high risk of impaired glucose regulation (*n* = 435) from the Walking Away from Diabetes randomized controlled trial, UK; cross-sectional	SB, standing, stepping – thigh-worn accelerometers; sleep – not assessed	Fasting glucose, 2-h glucose, fasting insulin, 2-h insulin, HOMA-IS, Matsuda-ISI	Mekary et al. [12] / 30 min	Age, sex, smoking status, family history of type 2 diabetes, ethnicity, β-blockers, lipid-lowering medication and activPAL waking wear time (model 1), and waist circumference (model 2).	β (95% CI) Model 1 Fasting glucose Prolonged SB → short SB: 1.00 (0.99, 1.00) Prolonged SB → standing: 1.00 (0.99, 1.01) Prolonged SB → stepping: 0.98 (0.97, 0.99) Short SB → standing: 1.00 (0.99, 1.01) Short SB → stepping: 0.99 (0.98, 1.00) 2-h glucose Prolonged SB → short SB: 0.99 (0.97, 1.01) Prolonged SB → standing: 1.00 (0.98, 1.01) Prolonged SB → stepping: 0.94 (0.92, 0.97) Short SB → standing: 1.00 (0.99, 1.00) Short SB → stepping: 0.95 (0.92, 0.97) Fasting insulin Prolonged SB → short SB: 0.96 (0.93, 0.99) Prolonged SB → standing: 0.95 (0.92, 0.98) Prolonged SB → stepping: 0.89 (0.84, 0.95) Short SB → standing: 0.98 (0.95, 1.01) Short SB → stepping: 0.93 (0.88, 0.98) 2-h insulin Prolonged SB → short SB: 0.97 (0.92, 1.02) Prolonged SB → standing: 0.94 (0.90, 0.99) Prolonged SB → stepping: 0.85 (0.78, 0.92) Short SB → standing: 0.98 (0.94, 1.02) Short SB → stepping: 0.87 (0.81, 0.93) HOMA-IS Prolonged SB → short SB: 1.04 (1.01, 1.07) Prolonged SB → standing: 1.06 (1.02, 1.10) Prolonged SB → stepping: 1.15 (1.06, 1.26) Short SB → standing: 1.02 (0.99, 1.05) Short SB → stepping: 1.10 (1.03, 1.17) Matsuda-ISI Prolonged SB → short SB: 1.03 (0.99, 1.07) Prolonged SB → standing: 1.06 (1.01, 1.11) Prolonged SB → stepping: 1.22 (1.09, 1.35) Short SB → standing: 1.02 (0.99, 1.06) Short SB → stepping: 1.17 (1.07, 1.27) Model 2 Fasting glucose Prolonged SB → short SB: 1.00 (0.99, 1.01) Prolonged SB → standing: 1.00 (0.99, 1.01) Prolonged SB → stepping: 0.99 (0.98, 1.00) Short SB → standing: 1.00 (0.99, 1.01) Short SB → stepping: 1.00 (0.98, 1.01) 2-h glucose Prolonged SB → short SB: 0.99 (0.97, 1.01) Prolonged SB → standing: 1.00 (0.98, 1.02) Prolonged SB → stepping: 0.95 (0.92, 0.98) Short SB → standing: 1.00 (0.99, 1.00) Short SB → stepping: 0.95 (0.93, 0.98) Fasting insulin Prolonged SB → short SB: 0.96 (0.94, 0.98) Prolonged SB → standing: 0.96 (0.93, 0.99) Prolonged SB → stepping: 0.93 (0.88, 0.98) Short SB → standing: 0.99 (0.96, 1.02) Short SB → stepping: 0.96 (0.92, 1.00) 2-h insulin Prolonged SB → short SB: 0.97 (0.92, 1.02) Prolonged SB → standing: 0.95 (0.91, 1.00) Prolonged SB → stepping: 0.87 (0.80, 0.94) Short SB → standing: 0.99 (0.95, 1.03) Short SB → stepping: 0.90 (0.83, 0.98) HOMA-IS Prolonged SB → short SB: 1.04 (1.01, 1.07) Prolonged SB → standing: 1.04 (1.01, 1.07) Prolonged SB → stepping: 1.09 (1.01, 1.17) Short SB → standing: 1.00 (0.97, 1.03) Short SB → stepping: 1.05 (0.99, 1.11) Matsuda-ISI Prolonged SB → short SB: 1.03 (0.98, 1.08) Prolonged SB → standing: 1.05 (1.01, 1.09) Prolonged SB → stepping: 1.16 (1.05, 1.28) Short SB → standing: 1.01 (0.96, 1.05) Short SB → stepping: 1.12 (1.03, 1.22)
Ekblom-Bak et al. [51]	Adults (*n* = 836) from the Swedish Cardio Pulmonary bioImage Study, Sweden; cross-sectional	SB, LPA, MPA, VPA – waist-worn accelerometers; sleep – not assessed	Triglyceride levels, HDL, blood pressure and glucose levels.	Mekary et al. [12] / 1, 5, 10, 15, 20, 25, 30, 60, 90 and 120 min	Age, sex, education level, smoking habits, perceived psychological stress, energy intake and wear time.	OR (95% CI) 10-min reallocation High triglyceride levels SB → LPA: 0.97 (0.94, 0.99) SB → MPA: 0.91 (0.83, 0.99) SB → VPA: 0.51 (0.33, 0.79) Low HDL SB → LPA: 0.95 (0.92, 0.98) SB → MPA: 0.91 (0.82, 1.01) SB → VPA: 0.34 (0.18, 0.65) High blood pressure SB → LPA: 1.00 (0.97, 1.02) SB → MPA: 0.92 (0.85, 0.99) SB → VPA: 0.77 (0.58, 1.04) High glucose SB → LPA: 0.99 (0.97, 1.01) SB → MPA: 0.97 (0.91, 1.03) SB → VPA: 0.88 (0.73, 1.07)
Ekblom-Bak et al. [52]	Adults (*n* = 654) from the Swedish Cardio Pulmonary bioImage Study, Sweden; cross-sectional	SB, LPA, MVPA – waist-worn accelerometers; sleep – not assessed	Systolic and diastolic blood pressure, glucose, triglycerides, HDL	Mekary et al. [12] / 1, 5, 10, 15, 20, 25, 30, 60, 90 and 120 min	Sex, age, education, smoking, perceived psychosocial stress.	RR (95% CI) 30 min reallocation Fasting glucose SB → LPA: 0.998 (0.995, 1.001) SB → MVPA: 0.991 (0.983, 0.999) Fasting insulin SB → LPA: 0.970 (0.954, 0.987) SB → MVPA: 0.884 (0.844, 0.927) HOMA-IR SB → LPA: 0.969 (0.951, 0.987) SB → MVPA: 0.876 (0.832, 0.923) Fasting glucose (< 6.0 mmol·l-1) SB → LPA: 0.980 (0.961, 0.999) SB → MVPA: 0.894 (0.846, 0.945) Fasting glucose (≥ 6.0 mmol·l-1) SB → LPA: 0.937 (0.906, 0.969) SB → MVPA: 0.889 (0.818, 0.967) Reallocating 5 to 120 min from SB to MVPA was associated with positive effects HOMA-IR (for participants with lower waist circumferences) and across all time lengths for participants with higher waist circumferences. Reallocating 1 to 120 min from SB to LPA or MVPA was associated with positive effects on HOMA-IR levels (for participants with low fitness). Reallocating 1 to 120 min from SB to MVPA was associated with positive effects HOMA-IR levels (for participants with high levels of fitness). Reallocating 1 to 120 min from SB to LPA or MVPA was associated with positive effects on HOMA-IR levels (for participants with high glucose levels). Reallocating 1 to 120 min from SB to MVPA was associated with positive effects HOMA-IR levels (for participants with normal glucose levels).
Falconer et al. [53]	Adults with type 2 diabetes (*n* = 519) from the Early Activity in Diabetes study, UK; cross-sectional	SB, LPA, MVPA – waist-worn accelerometers; sleep – not assessed	HDL	Mekary et al. [12] / 30 min	Age, sex, ethnic group, IMD score, accelerometer wear time, relevant diabetes or lipid-lowering drugs.	β (95% CI) HDL SB bouts → SB nonbouts: 0.01 (− 0.02, 0.03) SB bouts → LPA: 0.02 (0.01, 0.03) SB bouts → MVPA: 0.03 (− 0.01, 0.08) SB nonbouts → LPA: 0.01 (− 0.01, 0.04) SB nonbouts → MVPA: 0.03 (− 0.02, 0.07) LPA → MVPA: 0.02 (− 0.03, 0.06)
Hamer et al. [56]	Adults (*n* = 445) from the Whitehall II epidemiological cohort, UK; cross-sectional	SB, LPA, MVPA – waist-worn accelerometers; sleep – not assessed	HDL-C, triglycerides, HbA1c	Mekary et al. 2009 / 10 min	Age, sex, smoking, employment grade, and current statin use.	β (95% CI) HbA1c SB ↔ LPA: 0.001 (0.006, − 0.009) SB ↔ MVPA: − 0.023 (− 0.043, − 0.002) LPA ↔ MVPA: − 0.024 (− 0.047, − 0.001) HDL-C SB ↔ LPA: 0.005 (− 0.001, 0.01) SB ↔ MVPA: 0.037 (0.021, 0.054) LPA ↔ MVPA: 0.032 (0.014, 0.050) Triglycerides SB ↔ LPA: − 0.004 (− 0.014, 0.006) SB ↔ MVPA: − 0.035 (− 0.061, − 0.009) LPA ↔ MVPA: − 0.031 (− 0.060, − 0.002)
Healy et al. [58]	A general population-based sample (*n* = 698) from the 2011/12 Australian Diabetes, Obesity, and Lifestyle Study, Australia; cross-sectional	SB, standing, stepping – thigh-worn accelerometers; sleep – self report	Fasting glucose, HDL, LDL, total/HDL-cholesterol ratio, triglycerides and 2-h plasma glucose.	Mekary et al. [12] / 120 min	Age, sex, contraceptive pill use, blood pressure tablets, cholesterol tablets, diabetes medication, ethnicity, occupation and employment status, income, fibre intake, energy intake, energy-adjusted fibre intake, alcohol intake, sodium intake, potassium intake, fruit and vegetable serves.	RR (95% CI) Fasting glucose Sitting → standing: 0.98 (0.97, 1.00) Sitting → stepping: 0.98 (0.95, 1.02) Standing → stepping: 1.00 (0.97, 1.04) β (95% CI) HDL Sitting → standing: 0.06 (0.02, 0.09) Sitting → stepping: 0.10 (0.02, 0.18) Standing → stepping: 0.04 (− 0.05, 0.14) RR (95% CI) Total/HDL-cholesterol ratio Sitting → standing: 0.94 (0.92, 0.97) Sitting → stepping: 0.97 (0.92, 1.03) Standing → stepping: 1.04 (0.97, 1.11) RR (95% CI) Triglycerides Sitting → standing: 0.90 (0.87, 0.94) Sitting → stepping: 0.88 (0.78, 0.98) Standing → stepping: 0.98 (0.86, 1.11) RR (95% CI) 2-h plasma glucose Sitting → standing: 0.99 (0.96, 1.02) Sitting → stepping: 0.89 (0.84, 0.94) Standing → stepping: 0.90 (0.84, 0.97)
Healy et al. [57]	Individuals with diagnosed type 2 diabetes (*n* = 279) from the Living Well with Diabetes intervention, Australia; cross-sectional	SB (prolonged SB and non-prolonged SB), LPA, MVPA – waist-worn accelerometers; sleep – not assessed	Fasting plasma glucose	Mekary et al. [12] / 30 min	Age, sex, BMI, waist circumference, log HbA1c, insulin use, oral hypoglycaemic use, use of glucagon-like-peptide-1 agents, diabetes duration, income, education, weight loss aids in last 6 months, smoking status, CVD-related condition, musculoskeletal condition, depression and/or anxiety, employment, place of birth, Caucasian, energy intake, diet quality score.	RR (95% CI) Fasting blood glucose Prolonged SB → non-prolonged SB: 1.01 (0.99, 1.03) Prolonged SB → LPA: 0.99 (0.97, 1.00) Prolonged SB → MVPA: 0.96 (0.90, 1.03) Non-prolonged SB → LPA: 0.98 (0.96, 1.00) Non-prolonged SB → MVPA: 0.96 (0.89, 1.02) LPA → MVPA: 0.98 (0.91, 1.05)
Moore et al. [28]	Youth (*n* = 11,588) from the International Children’s Accelerometry Database, Brazil, Europe, and USA; cross-sectional	SB, LPA, MPA, VPA – waist-worn accelerometers; sleep – not assessed	Diastolic blood pressure, systolic blood pressure, HDL, LDL, glucose, insulin, triglycerides	Mekary et al. [12] / not presented	Accelerometer cut points.	Reallocating LPA with VPA was inconsistently related to blood pressure, fasting triglycerides, HDL, or LDL with only 32 of a possible 360 associations statistically significant. Reallocating LPA with VPA was associated with 12.6 to 27.0 pmol/l lower insulin values at the 75th to 90th percentiles.
Nilsson et al. [29]	Older woman (*n* = 113) recruited from an newspaper ad, Sweden; cross-sectional	SB (accumulated, continuous), LPA, MVPA – waist-worn accelerometers; sleep – not assessed	Triglycerides, HDL, plasma glucose, and blood pressure	Mekary et al. [12] / 10 min	Medical history, self-rated health status, total energy intake, fat intake, and alcohol consumption.	β (95% CI) Clustered metabolic risk score MVPA → LPA: 0.06 (0.01, 0.10) MVPA → accumulated SB: 0.07 (0.02, 0.11) MVPA → continuous SB: 0.08 (0.04, 0.13) Clustered metabolic risk score adjusted for waist circumference MVPA → LPA: 0.02 (− 0.02, 0.07) MVPA → accumulated SB: 0.04 (− 0.01, 0.09) MVPA → continuous SB: 0.05 (0.01, 0.10)
Rosique Esteban et al. [32]	Adults (*n* = 5776) from the PREDIMED-PLUS trial, Spain; cross-sectional	Sleep, SB, LPA, MVPA – self report	Blood pressure, hyperglycaemia, hypertriglyceridemia, HDL	Mekary et al. [12] / 60 min	Age, sex, education, marital and employment status, smoking habits, personal and family history of illness, medical conditions, medication use, and adherence to an energy-restricted Mediterranean diet.	RR (95% CI) Blood pressure MVPA → sleep: 0.99 (0.98, 1.00) MVPA → TV-viewing: 1.01 (0.99, 1.03) MVPA → LPA: 0.99 (0.98, 1.00) LPA → sleep: 1.00 (0.99, 1.01) LPA → TV-viewing: 1.00 (0.99, 1.01) Sleep → TV-viewing: 0.99 (0.97, 1.01) Hyperglycaemia MVPA → sleep: 0.98 (0.95, 1.01) MVPA → TV-viewing: 0.98 (0.96, 1.00) MVPA → LPA: 0.96 (0.93, 0.99) LPA → sleep: 1.02 (0.99, 1.05) LPA → TV-viewing: 1.02 (0.99, 1.05) Sleep → TV-viewing: 1.00 (0.98, 1.02) Hypertriglyceridemia MVPA → sleep: 0.94 (0.89, 0.99) MVPA → TV-viewing: 0.94 (0.90, 0.98) MVPA → LPA: 0.95 (0.88, 1.02) LPA → sleep: 0.99 (0.93, 1.05) LPA → TV-viewing: 0.97 (0.91, 1.03) Sleep → TV-viewing: 0.98 (0.95, 1.01) Low HDL MVPA → sleep: 0.92 (0.87, 0.97) MVPA → TV-viewing: 0.94 (0.90, 0.98) MVPA → LPA: 0.92 (0.86, 0.98) LPA → sleep: 0.97 (0.90, 1.04) LPA → TV-viewing: 0.99 (0.93, 1.05) Sleep → TV-viewing: 1.03 (1.00, 1.06)
Van der Berg et al. [35]	Adults (*n* = 2213) from The Maastricht Study, Netherlands; cross-sectional	SB, standing, stepping – thigh-worn accelerometers; sleep – not assessed	HDL, total-to-HDL cholesterol ratio, triacylglycerol, 2 h post-load glucose, fasting insulin	Mekary et al. [12] / 30 min	Age, sex, level of education, smoking status, alcohol consumption, energy intake, mobility limitation, and prevalent cardiovascular disease	OR (95% CI) HDL SB → standing: 0.005 (0.00, 0.01) SB → stepping: 0.041 (0.03, 0.05) Standing → stepping: 0.036 (0.02, 0.05) Total-to-HDL cholesterol ratio SB → standing: 0.993 (0.99, 1.00) SB → stepping: 0.981 (0.97, 0.99) Standing → stepping: 0.988 (0.98, 1.00) Triacylglycerol SB → standing: 0.991 (0.98, 1.00) SB → stepping: 0.975 (0.96, 0.99) Standing → stepping: 0.984 (0.97, 1.00) 2 h post-load glucose SB → standing: 0.999 (1.00, 1.00) SB → stepping: 0.981 (0.97, 0.99) Standing → stepping: 0.982 (0.97, 0.99) Fasting insulin SB → standing: 0.987 (0.98, 1.00) SB → stepping: 0.970 (0.95, 0.99) Standing → stepping: 0.983 (0.96, 1.01)
Varela-Mato et al. [37]	Adult male heavy goods vehicle drivers (*n* = 87), from a transport company from the East Midlands, UK; cross-sectional	SB, standing and stepping (LPA, MVPA) – thigh-worn accelerometers; sleep – estimated from the accelerometers and matched with the participants’ daily log	Systolic blood pressure, Diastolic blood pressure, fasting glucose, triglycerides, HDL, LDL, total cholesterol	Mekary et al. [12] / 30 min	Age, ethnicity, education levels, shift pattern, smoking, alcohol intake, fruit and vegetable consumption and BMI.	β (95% CI) Workdays Systolic blood pressure SB → standing: 0.6 (− 0.6, 1.9) SB → LPA: − 1.9 (− 5.1, 1.3) SB → MVPA: − 1.1 (− 5.5, 3.3) SB → sleep: − 0.3 (− 0.4, 0.4) Diastolic blood pressure SB → standing: 0.6 (− 0.5, 1.7) SB → LPA: − 1.8 (− 4.7, 0.9) SB → MVPA: 0.1 (− 3.8, 4.0) SB → sleep: − 0.2 (− 0.5, 0.2) Fasting glucose SB → standing: − 0.01 (− 0.1, 0.1) SB → LPA: − 0.04 (− 0.3, 0.2) SB → MVPA: − 0.3 (− 0.6, 0.1) SB → sleep: − 0.04 (− 0.1, 0.02) Triglycerides SB → standing: 0.00 (− 0.1, 0.1) SB → LPA: 0.06 (− 0.2, 0.3) SB → MVPA: − 0.4 (− 0.8, 0.01) SB → sleep: 0.02 (− 0.01, 0.05) HDL SB → standing: − 0.02 (− 0.06, 0.01) SB → LPA: − 0.1 (− 0.2, − 0.01) SB → MVPA: 0.3 (0.1, 0.4) SB → sleep: − 0.01 (− 0.02, 0.01) LDL SB → standing: − 0.04 (− 0.2, 0.1) SB → LPA: 0.2 (− 0.1, 0.6) SB → MVPA: − 0.1 (− 0.6, 0.5) SB → sleep: − 0.02 (− 0.07, 0.02) Total cholesterol SB → standing: − 0.6 (− 0.07, 0.02) SB → LPA: 0.1 (− 0.2, 0.5) SB → MVPA: 0.05 (− 0.4, 0.6) SB → sleep: − 0.2 (− 0.07, 0.02) Non-workdays Systolic blood pressure SB → standing: − 0.3 (− 1.1, 0.5) SB → LPA: 0.3 (− 1.3, 1.9) SB → MVPA: − 0.01 (− 3.7,3.7) SB → sleep: − 0.6 (− 1.2, 0.1) Diastolic blood pressure SB → standing: − 0.5 (− 1.2, 0.2) SB → LPA: − 0.5 (− 1.9, 0.9) SB → MVPA: 0.3 (− 2.9, 3.6) SB → sleep: − 0.3 (− 0.9, 0.2) Fasting glucose SB → standing: 0.01 (− 0.07, 0.1) SB → LPA: 0.03 (− 0.1, 0.2) SB → MVPA: − 0.3 (− 0.7, 0.05) SB → sleep: − 0.00 (− 0.06, 0.06) Triglycerides SB → standing: − 0.03 (− 0.1,0.04) SB → LPA: 0.07 (− 0.07, 0.2) SB → MVPA: − 0.2 (− 0.5, 1.0) SB → sleep: − 0.02 (− 0.08, 0.04) HDL SB → standing: 0.02 (− 0.01,0.05) SB → LPA: − 0.05 (− 0.1, 0.01) SB → MVPA: 0.07 (− 1.0, 0.2) SB → sleep: 0.0 (− 0.02, 0.03) LDL SB → standing: − 0.08 (− 0.2,0.01) SB → LPA: 0.0 (− 0.2, 0.2) SB → MVPA: − 0.01 (− 0.5, 0.4) SB → sleep: − 0.04 (− 0.2, 0.04) Total cholesterol SB → standing: − 0.08 (− 0.2,0.02) SB → LPA: − 0.03 (− 0.2, 0.1) SB → MVPA: 0.03 (− 0.4, 0.5) SB → sleep: − 0.03 (− 0.1, 0.04)
Wang et al. [74]	Individuals (*n* = 1699) with diabetes mellitus from the Hispanic Community Health Study/Study of Latinos (2008–2011), USA; cross-sectional	SB, LPA, MVPA – waist-worn accelerometers; sleep – not assessed	Blood pressure, HbA1c, LDL, HDL, and triglycerides.	Mekary et al. [12] / 30, 60 min	Age, sex, annual household income, education, employment status, Hispanic/Latino background, field centre, smoking, alcohol consumption, duration of Diabetes mellitus, health insurance status, alternative health eating index-2010, self-reported physical health score, and use of antidiabetic, antihypertensive, and lipid-lowering medications.	OR (95% CI)30 min HbA1c SB → MVPA: 1.06 (0.89, 1.25) Blood pressure SB → MVPA: 1.27 (1.04, 1.55) LDL SB → MVPA: 0.94 (0.79, 1.12) HDL SB → MVPA: 0.95 (0.81, 1.13) Triglycerides SB → MVPA: 0.91 (0.76, 1.09) 60 min HbA1c SB → LPA: 1.18 (1.04, 1.35) Blood pressure SB → LPA: 1.03 (0.91, 1.18) LDL SB → LPA: 1.04 (0.89, 1.22) HDL SB → LPA: 1.17 (1.04, 1.32) Triglycerides SB → LPA: 1.20 (1.05, 1.36)
Whitaker et al. [38]	Adults (*n* = 3211) from the CARDIA study, USA; cross-sectional	SB including [i] TV viewing, [ii] using the computer for non-work activities or playing video games, [iii] doing non-computer office work or paperwork, [iv] listening to music, reading a book or magazine, or doing arts and crafts, [v] talking on the phone or texting, and [vi] sitting in a car, bus, train or other mode of transportation, MVPA – self reports; LPA, sleep – not assessed	Composite cardio metabolic risk score	Mekary et al. [12] / 120 min	Age, sex, centre, race, education, unemployment, health insurance, smoking, alcohol, total physical activity, fast food and sugar sweetened beverage consumption, depressive symptoms and BMI.	β (95% CI) Composite cardio metabolic risk score TV viewing ↔ computer usage: − 0.07 (− 0.11, − 0.03) TV viewing ↔ paperwork: − 0.07 (− 0.12, − 0.02) TV viewing ↔ reading: 0.06 (− 0.11, − 0.02) TV viewing ↔ talking on the phone: − 0.07 (− 0.12, − 0.02) TV viewing ↔ sitting in a car: − 0.09 (− 0.13, − 0.05) Computer usage ↔ paperwork: − 0.00 (− 0.06, 0.06) Computer usage ↔ reading: 0.01 (− 0.04, 0.05) Computer usage ↔ talking on the phone: − 0.00 (− 0.06, 0.05) Computer usage ↔ sitting in a car: − 0.02 (− 0.06, 0.02) Paperwork ↔ reading: 0.01 (− 0.05, 0.06) Paperwork ↔ talking on the phone: − 0.00 (− 0.06, 0.06) Paperwork ↔ sitting in a car: − 0.02 (− 0.07, 0.04) Reading ↔ talking on the phone: − 0.01 (− 0.07, 0.05) Reading ↔ sitting in a car: − 0.03 (− 0.07, 0.02) Talking on the phone ↔ sitting in a car: − 0.02 (− 0.07, 0.04)
Yates et al. [76]	Adults at increased risk of type 2 diabetes (*n* = 508) from the Walking Away from Type 2 Diabetes Study, UK; cross-sectional	SB, LPA, MVPA – waist-worn accelerometers; sleep – not assessed	Fasting and 2-h post-challenge insulin and glucose, HOMA-IS and Matsuda-ISI	Mekary et al. [12] / 30 min	Age, sex, smoking status, beta-blocker and statin medication status, IMD score (model 1) and BMI (model 2).	OR (95% CI) Model 1 Fasting glucose SB → LPA: 1.00 (0.99, 1.01) SB → MVPA: 1.00 (0.98, 1.01) Fasting insulin SB → LPA: 0.98 (0.95, 1.01) SB → MVPA: 0.87 (0.81, 0.93) 2-h glucose SB → LPA: 0.97 (0.95, 0.99) SB → MVPA: 0.98 (0.95, 1.02) 2-h insulin SB → LPA: 0.96 (0.92, 1.00) SB → MVPA: 0.84 (0.76, 0.92) HOMA-IS SB → LPA: 1.02 (0.98, 1.05) SB → MVPA: 1.15 (1.07, 1.25) Matsuda-ISI SB → LPA: 1.05 (1.01, 1.09) SB → MVPA: 1.18 (1.08, 1.29) Model 2 Fasting glucose SB → LPA: 1.00 (0.99, 1.01) SB → MVPA: 1.00 (0.98, 1.02) Fasting insulin SB → LPA: 0.99 (0.96, 1.02) SB → MVPA: 0.92 (0.86, 0.99) 2-h glucose SB → LPA: 0.97 (0.95, 0.99) SB → MVPA: 0.98 (0.94, 1.02) 2-h insulin SB → LPA: 0.96 (0.91, 1.00) SB → MVPA: 0.85 (0.77, 0.94) HOMA-IS SB → LPA: 1.01 (0.97, 1.04) SB → MVPA: 1.08 (1.01, 1.16) Matsuda-ISI SB → LPA: 1.04 (1.00, 1.08) SB → MVPA: 1.14 (1.04, 1.25)

*SB* sedentary behaviour, *LPA* light intensity physical activity, *MVPA* moderate-to-vigorous intensity physical activity, *MPA* moderate intensity physical activity, *VPA* vigorous intensity physical activity, *BMI* body mass index, *HDL* high-density lipoproteins, *LDL* low-density lipoproteins, *HOMA-S* homeostasis model assessment of insulin sensitivity, *HOMA*-*β* homeostasis model assessment of β-cell function, *CVD* cardiovascular disease, *Mastuda-ISI* matsuda-insulin sensitivity index, *HbA1c* glycated haemoglobin, *RR* relative risk, *CI* confidence interval, *OR* odds ratio

**Table 7:** Summary of the findings from studies that assessed chronic diseases and conditions

Study	Sample	Measures of sleep, SB, LPA, MVPA	Outcome measures	Data analysis method / reallocated time	Adjustments for confounding	Results
Boeke et al. [46]	Women (*n* = 75,669) from the Nurses’ Health Study II, USA; prospective cohort	LPA, MPA, VPA – self-report; sleep, SB – not assessed	Breast cancer	Mekary et al. [12] / not presented	Age, height, age at menarche, oral contraceptive history, reproductive history, alcohol intake, body size at ages 10 and 20 years, weight, new pregnancies, breastfeeding duration, oral contraceptive and postmenopausal hormone use, menopausal status, age at menopause, and benign breast disease diagnosis.	No individual values were reported.
Ekblom-Bak et al. [51]	Adults (*n* = 836) from the Swedish Cardio Pulmonary bioImage Study, Sweden; cross-sectional	SB, LPA, MPA, VPA – waist-worn accelerometers; sleep – not assessed	Metabolic syndrome prevalence	Mekary et al. [12] / 1, 5, 10, 15, 20, 25, 30, 60, 90 and 120 min	Age, sex, education level, smoking habits, perceived psychological stress, energy intake and wear time.	OR (95% CI) 10-min reallocation Metabolic syndrome SB → LPA: 0.96 (0.93, 0.98) SB → MPA: 0.89 (0.82, 0.97) SB → VPA: 0.42 (0.26, 0.67) Reallocating 1 to 120 min from SB to LPA or MPA was associated with a decrease in metabolic syndrome prevalence. Reallocating 1 to 60 min from SB to VPA was associated with a decrease in metabolic syndrome prevalence.
Pinto et al. [30]	Adults with an increased risk for developing knee osteoarthritis (*n* = 1794) from the sub-cohort of the Osteoarthritis Initiative, USA; prospective cohort	SB, LPA, MVPA – waist-worn accelerometers; sleep – not assessed	Quality-adjusted life year	Mekary et al. [12] / 60 min	Age, sex, race/ethnicity, education, marriage status, income and BMI.	Reallocating 60 min from SB to MVPA was associated with an improvement in the quality-adjusted life year. Reallocating 60 min from SB to LPA did not result in any significant changes.
Rosique-Esteban et al. [32]	Adults (*n* = 5776) from the PREDIMED-PLUS trial, Spain; prospective cohort	Sleep, SB, LPA, MVPA – self report	Prevalence of type 2 diabetes	Mekary et al. [12] / 60 min	Age, sex, education, marital and employment status, smoking habits, personal and family history of illness, medical conditions, medication use, and adherence to an energy-restricted Mediterranean diet.	RR (95% CI) Type 2 diabetes MVPA → sleep: 0.95 (0.89, 1.01) MVPA → TV-viewing: 0.91 (0.86, 0.96) MVPA → LPA: 0.92 (0.85, 0.99) LPA → sleep: 1.03 (0.96, 1.10) LPA → TV-viewing: 1.00 (0.93, 1.07) Sleep → TV-viewing: 0.96 (0.93, 0.99)
Ryan et al. [33]	Adults (*n* = 2313) from the 2008 Health Survey for England, UK; prospective cohort	SB, LPA, MVPA – waist-worn accelerometers; sleep – not assessed	Chronic musculoskeletal pain	Mekary et al. [12] / 10, 30 min	Age, sex, BMI, socioeconomic status, diet, smoking history, alcohol intake, anxiety/depression, and presence of a non-musculoskeletal long-standing illness.	PR (95% CI) SB → LPA: 1.01 (0.99, 1.02) SB → MVPA: 0.90 (0.82, 0.98) In the secondary analysis reallocating 30 min of SB to MVPA resulted in relative risk reduction of 29%.
Wellburn et al. [75]	Adults (*n* = 1327) from the 2008 Health Survey for England, UK; prospective cohort	SB, LPA, MVPA – waist-worn accelerometers; sleep – not assessed	Prevalence of cardiovascular disease	Mekary et al. [12] / 10, 20 min	Age, sex, smoking status, socioeconomic status, diet, alcohol intake, anxiety/depression, musculoskeletal medication. Model 1 was adjusted for age alone, model 2 for age and sex and model 3 for all covariates.	RR (95% CI) 10 min Model 1 SB → LPA: 0.97 (0.95, 0.98) SB → MVPA: 0.89 (0.82, 0.96) Model 2 SB → LPA: 0.97 (0.96, 0.99) SB → MVPA: 0.87 (0.81, 0.94) Model 3 SB → LPA: 0.97 (0.96, 0.99) SB → MVPA: 0.88 (0.81, 0.96) 20 min SB → LPA: 0.95 (0.92, 0.98)

*SB* sedentary behaviour, *LPA* light intensity physical activity, *MVPA* moderate-to-vigorous intensity physical activity, *MPA* moderate intensity physical activity, *VPA* vigorous intensity physical activity, *BMI* body mass index, *OR* odds ratio, *CI* confidence interval, *RI* relative risk, *PR* prevalence ratio

### Methodological quality

The average score on the Newcastle-Ottawa scale for cross-sectional studies was 6, while the individual scores ranged from 5 to 8 points. Nine of the cross-sectional studies were classified as being of high quality, while 29 were appraised as being of moderate quality. The average score for prospective cohort studies was also 6, with the individual scores ranging from 4 to 8 points. Ten of the studies were classified as being of high quality, and the remaining ten were classified as being of moderate methodological quality. The methodological quality appraisal result for cross-sectional and prospective cohort studies can be found in Additional files 2 and 3, respectively.

## Discussion

This is the first review of health outcomes associated with reallocations of time between movement-related behaviours, compiling available evidence for all previously studied health outcomes across all age groups. The number of publications using ISM for health outcomes is increasing each year. Most commonly studied health outcomes in relation to isotemporal substitutions were mortality, general health, mental health, adiposity, fitness, and cardiometabolic biomarkers. The Mekary et al. [12] model has been by far the most often used method for investigating health outcomes of isotemporal substitution, but it is has also been available for the longest period of time. Findings from studies using ISM are of great value as they contribute evidence for the construction of meaningful recommendations for increasing physical activity, reducing sedentary behaviour, and optimizing sleep duration, by taking into account all the behaviours across the energy expenditure spectrum, and not just focusing on one behaviour in isolation from the others.

### Mortality

The current body of literature suggests that high sedentary time and low MVPA are predictive of premature mortality [8, 77]. When analysing data from the 2003–2006 US National Health and Nutrition Examination Survey (NHANES), Fishman et al. [54] concluded that reallocating 30 min/day of sedentary time with LPA reduced mortality risk by 20% over a five-year follow-up period. One study reported a 13% reduction in mortality risk when substituting 30 min/day of sedentary time with LPA [65]. The study found that replacement of the same amount of sedentary time with MVPA would reduce the mortality risk by as much as 81%. In addition to total volume, it seems therefore that physical activity intensity may play an important role in reducing the risk of premature mortality.

In a large-scale epidemiological study, Stamatakis et al. [72] reported beneficial associations on reducing the mortality risk when total sitting time was replaced with standing, walking, or MVPA. The study also considered sleep duration and indicated that reallocating 60 min/day of sedentary time to sleep may reduce the risk of mortality by 6%. However, this was found to be relevant only among individuals that reported sleeping fewer than seven hours per day.

When reallocating sedentary time to physical activity, Matthews et al. [66] reported a greater reduction in mortality risk for less active (< 2 h of total activity per day), than for more active (≥ 2 h of total activity per day) individuals. Follow-up work from the same group found that replacing one hour of sedentary time with LPA reduced mortality risk by 18% [67]. The reduction in mortality risk was more than doubled (42%) when sedentary time was reallocated to MVPA. In their analysis of the UK Biobank data, Wijndaele et al. [39] found that the substitution of 30 min/day of screen time with strenuous sports was associated with 15% reduction in the mortality risk. While in this study strenuous sports provided the most substantial associations, it is important to note that replacing screen time with 30 min/day of walking, also resulted in a considerable decrease in mortality risk (5%). Schmid et al. [71] reported that replacing 30 min/day of sedentary time with an equal amount of LPA or MVPA is associated with a 14 and 50% reduction in mortality risk, respectively. The same authors also found that replacing 30 min/day of sedentary time with LPA resulted in an 8% reduction in the risk of cancer mortality. The plausibility of these large estimated reductions in mortality risk needs to be confirmed in future studies.

Most of the evidence is currently focused on reallocations of time from sedentary behaviour to some intensity band of physical activity (e.g., LPA or MVPA). However, there is a lack of evidence on reallocating time spent in sedentary behaviour to standing. This might be because most devices for tracking physical activity, such as waist-worn accelerometers, are not able to differentiate between sedentary behaviour and standing. Furthermore, most questionnaires do not include questions regarding posture during certain types of typically sedentary activities, such as watching TV and inactive transport [1]. It is, therefore, unclear whether participants sat or stood while engaging in such activities. Nonetheless, one study suggested that replacing sedentary behaviour with standing might reduce the risk of premature mortality [72]. More such research seems to be needed to inform evidence-based decisions on the use of sit-stand desks and other methods for displacement of sitting with standing in the office setting. Given the increasing popularity of sit-stand desks, findings from these studies may have significant practical implications.

To summarise, it seems that reallocations of sedentary behaviour to LPA or MVPA are associated with significant reduction in mortality risk. Current evidence seems to consistently suggest that reductions in mortality risk are greater when time spent sedentary is replaced with higher intensities of physical activity. However, the generalizability of these findings remains limited, because the studies were conducted only in Australia [72], UK [39], and US [50, 54, 62, 65–67, 71]. There is no evidence for low and middle-income countries. Furthermore, most studies did not include sleep duration in their analysis. Due to the significant impact of sleep duration on health and its co-dependence on other components of time use, future studies should aim to include sleep duration in their ISMs along with all the remaining movement behaviours.

### Perceived/general health status

Studies that assessed perceived/general health status were conducted among adults, older adults, and clinical populations. No studies were conducted among youth.

#### Adults/older adults

Health-related quality of life (HRQoL) is an indicator of general health emanating from the individual perception of the impact that diseases have on different areas of life [45]. Loprinzi and Loenneke [65] investigated the impact of time reallocation between movement behaviours on HRQoL among adults using data from 2003 to 2006 NHANES. The study reported that substituting sedentary behaviour with LPA or MVPA was associated with improved HRQoL, suggesting the need for strategies to promote increasing physical activity at the expense of sedentary behaviour. Further support for such findings comes from Buman et al. [47] who found that replacing sedentary time with equal amounts of LPA or MVPA was associated with better self-reported physical health.

#### Clinical populations

Among clinical populations, Van Roekel et al. [73] reported improvements in some aspects of HRQoL to be associated with the replacement of sedentary time with standing, LPA or MVPA in a group of colorectal cancer survivors. Vallance et al. [34] investigated these relationships in a group of non-Hodgkins lymphoma survivors and found that reallocating sedentary time, sleep, or LPA to MVPA was associated with lower levels of fatigue, but not with improved quality of life. Studies like the one performed by Vallance et al. [34] are needed as non-Hodgkin lymphoma survivors and many other clinical populations exhibit higher levels of fatigue and poorer general health compared to non-clinical populations [78], and participating in physical activity appears to improve these health aspects [79].

While there are several studies exploring aspects of perceived general health using ISM, more evidence is needed on this topic. This lack of empirical evidence opens up an avenue for future studies, specifically studies including older adults and clinical populations. Only two of the included studies that assessed perceived general health included sleep in their ISMs, which is a key limitation of the current body of evidence.

### Mental health

Studies that assessed mental health were conducted among youth, adults, and older adults. No studies were conducted among clinical populations.

#### Youth

Janssen [60] investigated the associations of reallocating time between playing sedentary video games, playing active video games, and engaging in outdoor activities with mental health indicators among youth (mean age: 14.1 years; range 13.9–14.4 years). The results indicated that replacing time spent playing sedentary video games with playing active video games may be associated with more positive psychological outcomes, including indicators such as emotional problems, life satisfaction, and prosocial behaviours [60]. It should be noted that replacing active outdoor play with active video games was associated with the probability of high emotional problems, a reduced probability of high prosocial behaviour and high life satisfaction. The study supports active video games over sedentary video games; however, for the most positive mental health results, time spent on video games should be replaced with active outdoor play [60].

#### Adults/older adults

Depressive disorders are one of the most common mental health issues and affect around 10% of the adult population in the USA [80]. Previous studies have suggested that physical inactivity may be a risk factor for depressive symptoms [81]. Mekary et al. [69] reported that reallocating time spent watching TV to walking at a fast pace may be protective against depression. However, this was not found for reallocating time to walking at a slower pace, reinforcing the potential importance of higher intensities of physical activity. Rethorst et al.’s [31] study supports the importance of physical activity intensity as they noted that replacing sedentary time with VPA was associated with a significant decrease in depressive symptoms. However, the same was not observed for replacing sedentary time with LPA or MPA. Due to the scarcity of studies using ISMs while assessing mental health outcomes, further evidence on the topic is warranted.

### Adiposity

Studies assessing adiposity were conducted among youth, adults, older adults, and clinical individuals.

#### Youth

Adiposity was the most common outcome assessed in the studies included in this review. Aggio et al. [44] found that reallocating sedentary time with MVPA was associated with a significant reduction in body fat percentage in children and adolescents (age range: 5–15 years). Further evidence among children was presented by Collings et al. [21] who found that substituting sedentary time with LPA or MVPA was inversely associated with fat mass index and trunk fat mass index. Moreover, this study reported that the association was stronger when sedentary time was substituted with vigorous physical activity (VPA) than with MVPA. Similar findings were obtained among children in Norway [23], Sweden [27, 63], Portugal [70], and the UK [25]. Coupled with the conclusions presented in a recent meta-analysis [18], this evidence highlights the potential importance of substituting sedentary time with physical activity, in particular physical activity at vigorous intensities, to offset unhealthy fat gain in children.

#### Adults/older adults

The associations of ISM with adiposity were also explored in healthy adult populations. Buman et al. [48] reported a 2.4% lower waist circumference expected when 30 min/day of sedentary time were replaced with MVPA. Similar observations were made by Gupta et al. [55], who reported significant reductions in multiple obesity indicators, including waist circumference, body fat percentage and body mass index (BMI), associated with reallocating time from sedentary behaviour to standing time or MVPA, with the associations being greater for substitutions with MVPA. The stronger associations observed for substitution of sedentary behaviour with MVPA than with standing are likely because there is not a large difference in energy expenditure between sedentary behaviour and standing, unlike between sedentary behaviour and MVPA [82]. Beneficial associations between BMI and the reallocation of sedentary time to MVPA were also observed by Chastin et al. [13], who used a compositional model of isotemporal substitution based on the change-prediction matrix. Reallocating time from TV watching to MVPA was associated with a lower prevalence of obesity in Rosique-Esteban et al. [32].

#### Clinical populations

ISM has also been used to investigate how reallocations of time are associated with measures of adiposity among clinical populations. Boyle et al. [19] observed that reallocating time from either sleep, sedentary behaviour, or LPA to MVPA was associated with lower waist circumference and BMI in a group of breast cancer survivors. It is well established that MVPA is beneficial in the prevention and management of T2D [83]. Studies using accelerometry-derived behaviours found that the majority of individuals with T2D spent a significant portion of the day (63% of the day) in sedentary pursuits, with only 2% of the day spent in MVPA [84]. As reported by Healy et al. [57], reducing prolonged sedentary time and replacing it with MVPA was associated with lower waist circumference and BMI in individuals with T2D. Falconer et al. [53] found that even replacing prolonged bouts of sedentary time with shorter bouts of sedentary time was associated with lower BMI and waist circumference in adults with a recent diagnosis of T2D. While these findings would suggest that replacing sitting time with more ‘active’ behaviours might have significant health benefits in clinical populations, due to the cross-sectional nature of the studies, the causality of this relationship remains unclear.

In summary, it seems that reallocating sedentary time to physical activity may be associated with reduced BMI, body fat percentage and waist circumference in all populations, with the magnitude of associations being greater for higher intensity activities. However, most studies have not accounted for sleep time, which remains a major limitation in the current body of evidence. It is estimated that ~ 40% of the population reports sleeping less than the recommended minimum of seven hours per night [85] which may negatively affect health [86]. Studies have indicated that short sleep duration is associated with increased risk of obesity among children and adults [6]. Given its co-dependence with sedentary behaviour, LPA, and MVPA, sleep should, when possible, be included in isotemporal substitution models. Further research should track sleep duration in addition to sedentary time and physical activity, to increase the robustness of the findings.

### Fitness

Studies assessing fitness were conducted among youth, adults, and older adults. No such studies were conducted among clinical populations. Higher levels of muscular strength, as well as general and cardiorespiratory fitness, are considered important health markers in children and adolescents [87, 88]. Higher cardiorespiratory fitness is associated with a lower risk of being overweight or obese in puberty [88]. In adolescents, higher muscular strength is associated with a lower risk of premature mortality [89], and participation in resistance training is associated with more favourable body composition in overweight and obese children and adolescents [90].

#### Youth

When exploring fitness levels among children, Leppänen et al. [63] found that the reallocation of sedentary behaviour to LPA or MVPA was associated with greater cardiorespiratory fitness, as assessed by the 20-m shuttle run test. The magnitude of the associations was larger when sedentary behaviour was replaced with MVPA, rather than LPA. In a cohort of 10-year-old children, replacing MVPA with any other movement behaviour (i.e., sleep, sedentary time, and LPA) was associated with lower levels of cardiorespiratory fitness [25]. Therefore, it seems that reallocating sedentary time to LPA or MVPA should be promoted among children.

#### Adults/older adults

At the other end of the age spectrum, in a cohort of aged Japanese woman (age range: 76–89 years), Kim [61] concluded that reallocating sedentary time to MVPA may improve performance in several measures of fitness, including usual and maximum gait speed, 5-chair sit-to-stand tests, and timed up-and-go test. In their analysis of data from the Maastricht Study, van der Velde et al. [36] found that replacing sedentary time with LPA was associated with higher cardio-respiratory fitness. The findings seem encouraging and of potentially great importance for individuals who may have difficulties engaging in MVPA. While reallocating sedentary time to LPA was reported to be beneficial for cardio-respiratory fitness, reallocating the same durations of time to MVPA was associated with somewhat greater associations [32]. Overall, studies suggest likely improvements in fitness when time is reallocated from sedentary behaviour to physical activity. However, an additional evidence is needed to form firm conclusions, particularly among adult and clinical populations.

### Cardiometabolic biomarkers

Studies assessing cardiometabolic biomarkers were conducted among youth, adults, and older adults. There was no available evidence for clinical populations.

#### Youth

Only two studies thus far have used the ISM with cardiometabolic biomarkers as outcome variables [28, 49]. These studies used two different ISMs, and therefore, the comparability of their findings may be limited. A recent study compared the ISMs by Mekary et al. [12] and by Dumuid et al. [14] using the same dataset and reported differences in estimates between the models [91]. Using the model by Mekary et al. [12], Moore and colleagues [28] reported a beneficial associations of reallocating time from LPA to VPA with insulin levels. The association seemed stronger in individuals with higher baseline insulin levels. Carson et al. [49] employed the compositional ISM described by Chastin et al. [13] using data from the Canadian Health Measures Survey, with sleep, sedentary behaviour, LPA, and MPA as explanatory variables and systolic and diastolic blood pressure, C-reactive protein, HDL-cholesterol, triglycerides, and insulin level. The established associations were either unclear or practically insignificant. The cross-sectional design of both studies prevented drawing conclusions about causality of the established relationships. Future research is needed to strengthen the evidence base on the association between isotemporal substitutions and cardiometabolic markers in children and adolescents.

#### Adults/older adults

In their study using the 2005–2006 NHANES data, Buman et al. [48] investigated how reallocating time between sedentary behaviour, sleep, LPA, and MVPA was associated with waist circumference, systolic and diastolic blood pressure, high-density lipoprotein, C-reactive protein, low-density lipoprotein, plasma glucose, insulin, triglycerides, homeostasis model assessment of insulin sensitivity (HOMA-S), and homeostasis model assessment of β-cell function (HOMA-β). They found that reallocation of sedentary behaviour to MVPA was associated with increased levels of high-density lipoprotein cholesterol, and with a decrease in waist circumference, triglycerides, glucose, insulin, and HOMA-S [48]. Reallocating time from sedentary behaviour to LPA was beneficial for triglycerides, insulin sensitivity, and HOMA-β [48]. The study also considered sleep duration and reported that reallocating time from sedentary behaviour to sleep was beneficially associated with insulin, HOMA-S, HOMA-β. The same association was found for LDL, albeit only in long sleepers (≥ 8 h of sleep per night). Consistent with studies on other health outcomes, Buman et al. [48] inferred that the greatest benefits might be expected from increasing MVPA at the expense of other movement behaviours.

Edwardson et al. [24] reported that reallocation of prolonged sitting to standing or stepping improved 2-h glucose levels, fasting and 2-h insulin levels, and insulin sensitivity in individuals at high risk of impaired glucose regulation or T2D. While modest in terms of magnitudes of associations, the results seem promising for this population. Increasing habitual MVPA levels may be difficult for some individuals and population sub-groups. Evidence from other studies further supports the findings that reallocating sedentary time to LPA (standing and stepping) is associated with more favourable Matsuda-Insulin sensitivity index, levels of high-density lipoprotein [48], and glycosylated hemoglobin [53]. Overall, studies indicated that reallocating sedentary time to LPA or MVPA was associated with favourable cardiometabolic outcomes. While there is a relatively large body of evidence regarding associations between time reallocation and cardiometabolic biomarkers among adults, few studies have been conducted among children, adolescents, and older adults. In addition, the studies among older adults were conducted on small sample sizes [29]. Future studies should employ longitudinal study designs to further investigate associations of isotemporal substitution with fitness-related outcomes.

### Chronic diseases and conditions

Studies assessing chronic diseases and conditions were conducted among adults and older adults. There was no available evidence for clinical populations.

#### Adults/older adults

In total, six eligible studies were identified. The assessed outcomes included: cardiovascular disease, T2D, metabolic syndrome, breast cancer, chronic musculoskeletal pain, and quality-adjusted life years. Significant associations were found between reallocating time spent in sedentary behaviour to LPA or MVPA and a reduced risk of cardiovascular disease [75]. Furthermore, reallocation of time from TV-viewing to MVPA was associated with a significantly lower risk of T2D [32]. Ekblom-Bak et al. [51] noted that reallocating time from SB to LPA or MPA was associated with a decrease in the risk of metabolic syndrome. For breast cancer, Boeke et al. [46] found no evidence that substituting MVPA for walking was associated with breast cancer risk. Ryan et al. [33], reported a 29% relative risk reduction in chronic musculoskeletal pain when reallocating 10 min of sedentary behaviour to MVPA. Evidence on various other chronic disease and conditions is scarce. A study by Pinto and colleagues [30] assessing quality-adjusted life years indicated that reallocating 60 min from SB to MVPA was associated with a significant improvement in this outcome. The study did not find any evidence that reallocating SB to LPA was associated with quality-adjusted life years. More research is needed about the effects of isotemporal substitutions on chronic diseases and conditions.

### Statistical considerations and methodological quality

The ISM introduced by Mekary et al. [12] represents the first attempt within physical activity and sedentary behaviour research to deal with the constrained nature of time-use variables (i.e., the day only ever has 24 h for every participant), and to account for the fact that one time-use domain cannot be altered without compensatory changes in other time-use domains.

Subsequently, some researchers have suggested that time-use data are compositional data that occupy a constrained sample space and convey relative information [1, 2, 13, 14]. Accordingly, statistical methods designed for vectors in real space (including the isotemporal substitution model proposed by Mekary et al. [12], which may, therefore, not be appropriate for time-use data which do not occupy real space [1, 2, 13, 14]. The compositional isotemporal substitution (based on the change-prediction matrix) presented in Chastin et al. [13] and later used in Carson et al. [47] was the first attempt to apply the logic of the Mekary et al. [12] ISM method in a compositional data analysis framework. The compositional isotemporal approach was subsequently simplified by Dumuid et al. [14]. There is an ongoing discussion among researchers in the field of epidemiology about which of these methods should preferably be used [2, 14]. Interested readers can find detailed statistical reasoning about the potential implications of using each of the three methods elsewhere [14, 15].

It is important to highlight that from a methodological standpoint, all of the included studies were classified as being of high or moderate quality. The findings seem to be generally consistent across the studies, indicating that they were not significantly influenced by differences in study designs. A strength of most included studies is the use of device-based measures for assessing time spent in movement behaviours. Self-reports were used in some large-scale studies, which seems reasonable given accelerometry may significantly add to the administrative burden for researchers [92]. However, no systematic differences could be observed in the findings from studies relying on self-reports and device-based measures. The majority of the ISM studies used the linear regression model [21, 24, 27, 29, 35, 36, 39, 44–46, 48, 54, 56, 57, 63, 70, 72, 76]. Most of them [21, 24, 27, 29, 35, 36, 39, 44–46, 48, 54, 56, 57, 63, 70, 72, 76] assessed the linearity of the relationships prior to running the ISM analysis and indicated that they were linear. Using compositional data analysis, Dumuid et al. [14] have shown that associations of isotemporal substitutions with health outcomes may be non-linear and may depend on the reference composition. Future studies should consider these possibilities.

Furthermore, statistical techniques underpinning Mekary et al. [12] ISM, Chastin et al. [13] ISM, and Dumuid et al. [14] ISM are different; hence the findings of studies using different ISM models may not necessarily be directly comparable. Moreover, a recent study [91] indicated there may be substantial differences in estimates obtained from ISMs proposed by Mekary et al. [12] and Dumuid et al. [14]. It is, therefore, important for future studies to clearly specify which ISM model was used. It is important to note, however, that none of the three ISMs can mitigate standard limitations of studies in this area, such as poor measurement of the exposure, potential reverse causality, and unmeasured or poorly measured potential confounding variables.

The main limitation of the current body of evidence pertains to the low number of studies that included all daily movement-related behaviours (e.g., sedentary behaviour, sleep, LPA, and MVPA) in the ISMs. While studies did adjust for many confounding variables, only those that included all of the behaviours received a point on the item regarding adjustments for confounding on the Newcastle-Ottawa scale, as otherwise the adjustments would be considered insufficient [2]. Future studies should endeavour to include sedentary behaviour, sleep, LPA, and MVPA in their models. Most of the studies were cross-sectional, which is a major limitation of the current body of evidence on isotemporal substitution effects. While there were 18 prospective cohort studies included in this review, they cover only a limited range of outcomes. More longitudinal studies are needed to covering a wide range of health outcomes that are potentially associated with isotemporal substitutions.

## Conclusions

There is a notable increase in interest in the associations between time reallocation among movement-related behaviours and their relationship to health outcomes. While much media coverage has focused on the adverse effects of prolonged sedentary behaviour [93], evidence in this review suggests it would be more appropriate to shift the focus on the importance of reallocating time spent in sedentary behaviour to time spent in physical activity. The approach taken in this review broadens the physical activity behavioural arena, for both researchers and policymakers. While the current body of evidence indicates that time reallocation between sleep, sedentary behaviour, LPA, and MVPA may be associated with a number of health outcomes, there is a need for future studies employing longitudinal study designs, taking into account all movement behaviours, and examining a wider range of health, psychological, social, economic, and environmental outcomes. Future ISM studies on outcomes such as stroke, myocardial infarction, and cardiovascular disease death endpoints are warranted, given the current lack of evidence in this area.

## Additional files

Additional file 1: Search syntax. (DOCX 11 kb)

Additional file 2: Methodological quality appraisal of cross-sectional studies. (DOCX 18 kb)

Additional file 3: Methodological quality appraisal of prospective cohort studies. (DOC 15 kb)
