# Supplementary material for: Health outcomes associated with reallocations of time between sleep, sedentary behaviour, and physical activity: a systematic scoping review of isotemporal substitution studies
Source: Int J Behav Nutr Phys Act. 2018 Jul 13;15:69. doi: 10.1186/s12966-018-0691-3 (PMC6043964; doi:10.1186/s12966-018-0691-3)
Supplement: Supplementary file 1 — Search syntax. (DOCX 11 kb) [file 12966_2018_691_MOESM1_ESM.docx]

Supplementary file 1. Search syntax

Scopus:

title-abs-key("physical activity" or "physical inactivity" or sedentar* or sleep* or sitting or standing) and title-abs-key(isotemporal or compositional)

PubMed/MEDLINE:

("physical activity"[tw] OR "physical inactivity"[tw] OR sedentar*[tw] OR sleep* OR sitting[tw] or standing[tw]) AND (isotemporal[tw] OR compositional[tw])

Web of Science:

TS=("physical activity" or "physical inactivity" or sedentar* or sleep* or sitting or standing) AND TS=(isotemporal or compositional)

Other databases (through EBSCOhost):

("physical activity" or "physical inactivity" or sedentar* or sleep* or sitting or standing) AND (isotemporal or compositional)

Searches were performed in July 2017.
