# Supplementary material for: Health outcomes associated with reallocations of time between sleep, sedentary behaviour, and physical activity: a systematic scoping review of isotemporal substitution studies
Source: Int J Behav Nutr Phys Act. 2018 Jul 13;15:69. doi: 10.1186/s12966-018-0691-3 (PMC6043964; doi:10.1186/s12966-018-0691-3)
Supplement: Supplementary file 2 — Methodological quality appraisal of cross-sectional studies. (DOCX 18 kb) [file 12966_2018_691_MOESM2_ESM.docx]

Supplementary file 2. Methodological quality appraisal of cross-sectional studies

| Study | Representativeness of the sample | Sample size | Non-respondents | Ascertainment of the exposure (risk factor): | Comparability and adjustments for confounding | Assessment of the outcome | Overall score |
| --- | --- | --- | --- | --- | --- | --- | --- |
| Aggio et al. [44] | * | - | - | ** | - | ** | 5 |
| Boyle et al. [19] | * | - | * | ** | * | * | 6 |
| Buman et al. [48] | * | * | * | ** | * | ** | 8 |
| Carson et al. [49] | * | * | - | ** | * | ** | 7 |
| Chastin et al. [13] | * | * | - | ** | * | ** | 7 |
| Collings et al. [20] | * | * | - | ** | * | ** | 7 |
| Collings et al. [21] | * | - | - | ** | * | ** | 6 |
| Dahl-Petersen et al. [22] | * | * | - | ** | - | ** | 6 |
| Dalene et al. [23] | * | * | - | ** | - | ** | 6 |
| Edwardson et al. [24] | * | * | * | ** | - | ** | 7 |
| Ekblom-Bak et al. [51] | * | * | - | ** | - | ** | 6 |
| Ekblom-Bak et al. [52] | * | * | - | ** | - | ** | 6 |
| Fairclough et al. [25] | * | - | - | ** | * | ** | 6 |
| Falconer et al. [53] | * | * | - | ** | - | ** | 6 |
| Fanning et al. [26] | * | - | - | ** | * | ** | 6 |
| Gupta et al. [55] | * | * | - | ** | * | ** | 7 |
| Hamer et al. [56] | * | * | - | ** | - | ** | 6 |
| Healy et al. [57] | * | * | - | ** | * | ** | 7 |
| Healy et al. [58] | * | - | - | ** | - | ** | 5 |
| Janssen [60] | * | * | * | * | - | * | 5 |
| Kim [61] | * | - | - | ** | - | ** | 5 |
| Leppänen et al. [63] | * | * | * | ** | * | ** | 8 |
| Loprinzi et al. [65] | * | * | - | ** | - | ** | 6 |
| Moore et al. [28] | * | * | - | ** | - | ** | 6 |
| Nilsson et al. [29] | * | - | * | ** | - | ** | 6 |
| Rethorst et al. [31] | * | * | - | ** | - | * | 5 |
| Rosique-Esteban et al. [32] | * | * | * | ** | * | - | 6 |
| Ryan et al. [33] | * | * | - | ** | - | * | 5 |
| Sardinha et al. [70] | * | - | - | ** | - | ** | 5 |
| Vallance et al. [33] | * | - | - | ** | * | * | 5 |
| Van der Berg et al. [35] | * | * | - | ** | - | ** | 6 |
| Van der Velde et al. [36] | * | * | - | ** | - | ** | 6 |
| Van Roekel et al. [73] | * | - | * | ** | * | * | 6 |
| Varela-Mato et al. [37] | * | - | - | ** | * | ** | 6 |
| Wang et al. [74] | * | * | * | ** | - | ** | 7 |
| Wellburn et al. [75] | * | * | * | ** | - | * | 6 |
| Whitaker et al. [38] | * | * | - | * | - | ** | 5 |
| Yates et al. [76] | * | * | - | ** | - | ** | 6 |
| * = criteria met (1 point); ** = criteria met (2 points); - = criteria not met | | | | | | | |
