# Supplementary material for: Health outcomes associated with reallocations of time between sleep, sedentary behaviour, and physical activity: a systematic scoping review of isotemporal substitution studies
Source: Int J Behav Nutr Phys Act. 2018 Jul 13;15:69. doi: 10.1186/s12966-018-0691-3 (PMC6043964; doi:10.1186/s12966-018-0691-3)
Supplement: Supplementary file 3 — Methodological quality appraisal of prospective cohort studies. (DOC 15 kb) [file 12966_2018_691_MOESM3_ESM.docx]

Supplementary file 3. Methodological quality appraisal of prospective cohort studies

| Study | Representativeness of the exposed cohort | Selection of the non-exposed cohort | Ascertainment of exposure | Demonstration that outcome of interest was not present at start of study | Comparability of cohorts on the basis of the design or analysis | Assessment of outcome | Was follow-up long enough for outcomes to occur | Adequacy of follow-up of cohorts | Overall score |
| --- | --- | --- | --- | --- | --- | --- | --- | --- | --- |
| Balboa-Castillo et al. [45] | * | * | - | * | * | - | * | - | 5 |
| Boeke et al. [46] | * | * | - | * | - | * | * | * | 6 |
| Buman et al. [47] | * | * | * | * | - | - | * | * | 6 |
| Dalene et al. [23] | * | * | * | * | - | * | * | - | 6 |
| Chomistek et al. [50] | * | * | - | * | - | * | * | * | 6 |
| Fishman et al. [54] | * | * | * | * | - | * | * | * | 7 |
| Huang et al. [59] | * | * | * | * | * | * | * | * | 8 |
| Lee [62] | * | * | * | * | - | * | * | * | 7 |
| Leppänen et al. [27] | * | * | * | * | * | * | * | - | 7 |
| Loprinzi et al. [65] | * | * | * | * | - | * | * | * | 7 |
| Matthews et al. [66] | * | * | - | * | * | * | * | * | 7 |
| Matthews et al. [67] | * | * | * | * | - | * | * | * | 7 |
| Mekary et al. [12] | * | * | - | * | - | - | * | - | 4 |
| Mekary et al. [69] | * | * | - | * | - | - | * | * | 5 |
| Mekary et al. [68] | * | * | - | * | * | - | * | * | 6 |
| Pinto et al. [30] | * | * | * | * | - | - | * | - | 5 |
| Sardinha et al. [70] | * | * | * | * | - | * | * | - | 6 |
| Schmid et al. [71] | * | * | * | * | - | * | * | * | 7 |
| Stamatakis et al. [72] | * | * | - | * | * | * | * | * | 7 |
| Wijndaele et al. [39] | * | * | - | * | * | * | * | * | 7 |
| * = criteria met; - = criteria not met | | | | | | | | | |
